# The Tomato DOF Daily Fluctuations 1, *TDDF1* acts as flowering accelerator and protector against various stresses

**DOI:** 10.1038/s41598-017-10399-7

**Published:** 2017-08-31

**Authors:** Mohamed Ewas, Eman Khames, Khurram Ziaf, Raheel Shahzad, Elsayed Nishawy, Farhan Ali, Hizar Subthain, Mohamed H. Amar, Mohamed Ayaad, Omran Ghaly, Jie Luo

**Affiliations:** 1 National Key Laboratory of Crop Genetic Improvement and National Center of Plant Gene Research (Wuhan), College of Life Science and Technology, Huazhong Agricultural University, Wuhan Hubei, 430070 China; 2 Genetic Resources Department, Deserts Research Center (DRC), Cairo, Egypt; 3 College of Pharmacy, Tanta University, Tanta, Egypt; 4 Institute of Horticultural Sciences, University of Agriculture, Faisalabad, Pakistan; 5 Cereal Crops Research Institute (CCRI), Nowshera, Pakistan; 6 Egyptian Atomic Energy Nuclear Research Center, Inshas, Egypt

## Abstract

Adaptation to environmental changes is an important fitness trait for crop development. Photoperiod is an essential factor in seasonal control of flowering time. Sensing of day-length requires an interaction between the Photoperiod and the endogenous rhythms that is controlled by plant circadian clock. Thus, circadian clock is a critical regulator and internal molecular time-keeping mechanism, controlling key agricultural traits in crop plants such as the ability to adjust their growth and physiology to anticipate diurnal environmental changes. Here, we describe the gene *Tomato Dof Daily Fluctuations* 1 (*TDDF1*), which is involved in circadian regulation and stress resistance. Large daily oscillations in *TDDF1* expression were retained after transferring to continuous dark (DD) or light (LL) conditions. Interestingly, overexpressing *TDDF1* induce early flowering in tomato through up-regulation of the flowering-time control genes, moreover, by protein-protein interaction with the floral inducer *SFT* gene. Notably, overexpressing *TDDF1* in tomato was associated with chlorophyll overaccumulation by up-regulating the related biosynthetic genes. *TDDF1* expression results in improved drought, salt, various hormones stress tolerance alongwith resistance to late blight caused by *Phytophthora infestans*. This study can be a distinctive strategy to improve other economically important crops.

## Introduction

Tomato is one of the major economically important crops and is extensively used in different forms throughout the world. As many plants, tomato are using day length as an environmental clue to assure adequate timing of switch from vegetative to reproductive growth^1^. The main factors in seasonal control of flowering time are photoperiod and temperature as well as the response to these inputs, hence, the timing of flowering is controlled by the circadian clock^2^. DNA binding with One Finger (DOF) proteins is a group of plant-specific transcription factors (TFs) that contain a 50 aa conserved domain in the N-terminal region^3^. In Arabidopsis and tomato, DOF factors whose transcripts oscillate under constant light conditions and are hence known as Cycling DOF Factors, *CDF1*–5^3–5^. CDFs display an important role in photoperiodic flowering in Arabidopsis and tomato through the establishment of a diurnal rhythm in CONSTANS (CO) transcript levels by regulating its expression. When overexpressed, *CDF1*–5 repress CO transcription, causing a strong delay of flowering under long-day (LD) conditions. Consistently, combining loss-of-function alleles in four of these genes (*CDF1*, 2, 3, and 5) causes photoperiod-insensitive early flowering^5^. In tomato, SFT can act as both a flowering promoter and an activator of genes involved in the maintenance of the vegetative program^6^.

Naturally, the cultivated form of tomato is widely susceptible to several biotic and abiotic stresses. This is the prime reason of drastic reduction in yield and quality^7^. However, the wild relatives of tomato possess wide tolerance levels to different stresses^8^. Most of tomato cultivars show negative effects on germination, growth, and both quality and quantity of fruit under drought and salinity^9^. Drought and salinity significantly affect the process of photosynthesis which ultimately decease other important metabolic pathways^10^. Drought and salinity stress also affect the antioxidant and osmoregulation pathways that reinforce plant cells by the biosynthesis of compatible solutes and reactive oxygen species (ROS) scavengers^11–15^.

Drought is by far the most important environmental constrain in agriculture^16^ and every year the impact of economic losses increases dramatically due to water constraints^17^. Plants have evolved a stunning array of biochemical and physiological mechanisms to adapt the adverse environmental conditions^18^. A complex network of highly coordinated hormonal interactions seems to be crucial for manipulating the germplasm in desirable direction^19^. Classically, the role of phytohormones have been described considering individual signaling pathway but this approach does not address the spatiotemporal specificity, considered central to fine-tune hormone signaling. Thus, the study of hormone profiling of water stress and high salinity conditions could help to distinguish common from specific responses. Perception of stress signals often causes changes in levels of different hormones for adapting and responding to specific environmental challenges^20, 21^. Among phytohormones ethylene (EA), abscisic acid (ABA), jasmonic acid (JA) and salicylic acid (SA) are commonly known to play important roles in plant responses in stress condition. Classically, ABA has been mainly related to abiotic stress^22, 23^ while, JA and SA have been considered signals of biotic stress threats for years because they fulfill essential role in plant defense^24^. *NCED3*, *OPR3* and *PAL1* are known as key genes in ABA, JA and SA biosynthesis pathways, respectively^25–27^. Late blight (LB), caused by the oomycete *Phytophthora infestans* is one of the most destructive and devastating diseases of tomato worldwide. This disease is causing significant economic losses annually to the total income from this crop^28, 29^. Specific effectors can act as virulence (Avr) factors and activate corresponding host-plant resistance genes (R-genes) according to the gene-for-gene resistance model^30^. These genes can provide broad spectrum resistance to increase the economic importance of tomato and meet the global food demands. Over the last few years, DOF proteins have been reported to play a handy role in different biological processes, such as tissue specific gene expression, light responses, plant hormone signaling^31–34^, photoperiodic flowering^4, 35^, and flower abscission^36^ etc. DOF transcription factors are involved in multiple aspects of plant growth and development but their precise roles in biotic and abiotic stress tolerance are largely unknown^3^. Therefore, extensive work with high level of precision is required to identify the exact role of DOF transcription factors and manipulate the germplasm according to the need of humanity.

The ability of crop to confer multiple desirable traits in a single variety is highly demanded in this advance arena of molecular biology. This idea usually requires selection of multiple alleles of more than one gene for broad spectrum resistance against several adverse environmental conditions and biological hurdles^18^. Consequently, pyramiding all desirable genes by single breeding program or even genetic engineering is a cumbersome task and consuming a lot of resources. Therefore, plant scientist needs to address the bottlenecks of gene identification and role of different genes at the same time. In this report, we have achieved enticing traits such as improved drought, salt, various hormones stress tolerance and resistance to *Phytophthora infestans* alongwith early flowering by expressing a single gene coding for Tomato DOF Daily Fluctuations, 1 (*TDDF1*). This research also designed to facilitate and better understand the crosstalk between JA, SA, and ABA in tomato plants in response to drought and salt stress. The study appears to be of remarkable interest since *TDDF1* involved in circadian regulation and early flowering, and stress resistance.

## Results

### Structure of the *TDDF1* gene

Based on previous microarray results^37^, a differentially expressed drought-responsive Dof gene (SGN-U570634, http://solgenomics.net) was identified. The unigene information and full-length cDNA from KafTom (http://www.pgb.kazusa.or.jp/kaftom/) was successfully employed to design the gene amplification primers *TDDF1*-F/R (Table S1) which were used to obtain the full-length cDNA of *TDDF1* of size 1323 bp. A BLAST homology search of the tomato genome database (http://solgenomics.net/) revealed that the *TDDF1* gene was located on chromosome 5. *TDDF1* was predicted to encode a protein of size 441 amino acids, with a molecular weight of 49.1 kDa.

Phylogenetic analysis of a DNA binding with one finger in *Solanum pennellii*, *TDDF1*, a gene identified in the microarray analysis as one of the drought responsive genes^38^, predicted that *TDDF1* is structurally closely related to Dof family^37^ (Fig. S1). The phylogenetic result showed that *TDDF1* lies most close to *OsRdd1* in rice and *CDF1*, *CDF2* and *CDF3* in both tomato and Arabidopsis.

In an attempt to investigate traits that are regulated by *TDDF1*, the plasmid 35 S:*TDDF1* was introduced into cultivated tomato Ailsa Craig. Transgenic plants overexpressing (OE) *TDDF1* were obtained after screening of regenerated shoots on selection medium containing kanamycin. The transgenic plants were analyzed further by PCR using genomic DNA as template, and 35S forward and gene-specific reverse primers. Eighteen *TDDF1* OE lines were obtained as revealed by real-time PCR analyses. From these lines, three OE (OE1, OE4 and OE8) lines with a high transcript level were selected for further investigation (Fig. S2a).

### Expression pattern of *TDDF1* in tomato

**Figure 1:**
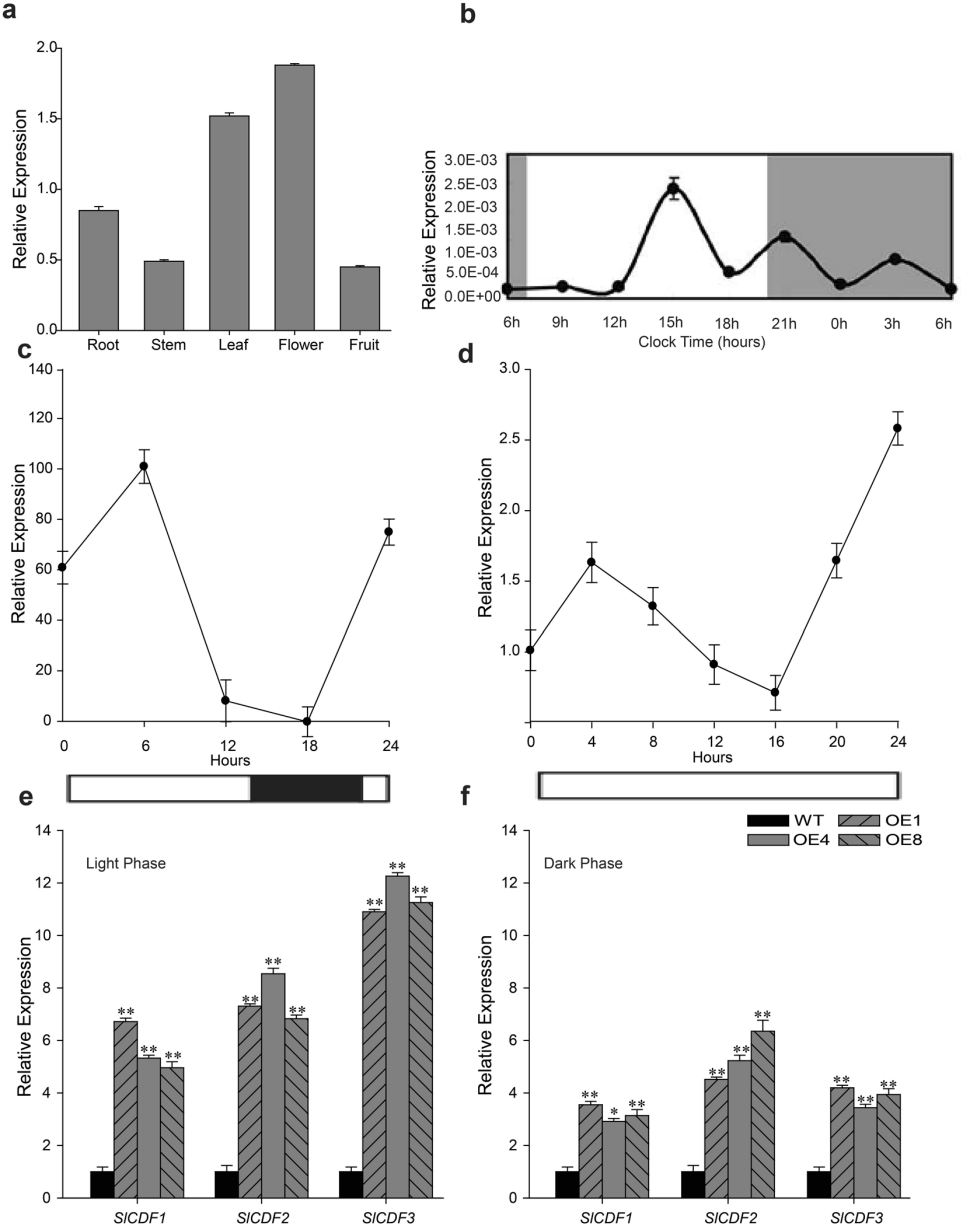
Expression patterns of *TDDF1*. (**a**) Tissue profiling analysis of *TDDF1* organs of cultivated tomato Ailsa Craig (*Solanum lycopersicum*) using qRT-PCR (relative to actin); (**b**) Expression pattern of *TDDF1* during a 24 h period (relative to actin). Leaf samples were collected every 3 h for 24 h starting from 06.00 h; (**c**,**d**) transcription analysis of *TDDF1* in response to different light conditions by qRT-PCR in 7-week-old tomato plants grown under a diurnal cycle of 16 h light/8 h dark or under continuous light. White and black bars along with the horizontal axis represent light and dark periods, respectively; (**e**,**f**) Expression levels of *SlCDF1*, *SlCDF2* and *SlCDF3* (circadian clock-regulated expression genes) in *TDDF1* OE and WT leaves by qRT-PCR under light and dark phase of L/D conditions. Single (*P < *0*.05) and double (**P < 0.01) asterisks denote statistically significant differences between the transgenic and wild-type lines.

To examine the spatial expression pattern of *TDDF1*, we conducted RT-PCR analysis with total RNA extracted from several tissues of the wild relative *S*. *pennellii* LA716 and in cultivated tomato *S*. *lycopersicum* Ailsa Craig, including young stems, leaves, flowers, fruits, and roots. In contrast to its homologue genes which preferentially express in root, tissue specific expression analysis revealed that *TDDF1* was abundant and expressed ubiquitously in all tissues tested with slightly high levels in flowers followed by leaves (Fig. 1a), suggesting its possible function out of root. Interestingly, during the day/night cycles, the *TDDF1* transcripts exhibited maximum expression in the afternoon followed by night expression, while the minimum expression was detected in the morning (Fig. 1b). In addition, the subcellular localization of *TDDF1* protein was determined and suggested that *TDDF1* was localized in the nucleus as expected for transcription factors (Fig. S2b). The promoter of *TDDF1* was cloned and analyzed. Some *cis*-acting regulatory elements involved in the light response, circadian control, and the phytohormone response, such as ABA, SA, and abiotic stress (e.g. MYB binding site), were found in the *TDDF1* promoter region (Fig. S3).

### Diurnal Rhythmic Expression Patterns of *TDDF1*

To further confirm whether the accumulation of *TDDF1* transcript also follow daily fluctuations round the clock or not, transcript of *TDDF1* was measured in the leaves of tomato plants grown under an L/D cycle regimen (16 h/8 h light/dark), and from those transferred from L/D to LL conditions. The *TDDF1* transcript exhibited large daily oscillations irrespective of light conditions, indicating that *TDDF1* expression was regulated by the circadian clock. Under LD conditions, expression level of *TDDF1* oscillated during the day and the peak was seen in the middle of the subjective light period and the trough in the subjective dark period (Fig. 1c). However, under LL conditions, the expression of *TDDF1* exhibited a 24 h period oscillation pattern and the peak was seen after 4 h while, the trough was seen after 16 h (Fig. 1d). Taken together, the dramatic difference in *TDDF1* expression between L/D and LL conditions indicated that *TDDF1* is light responsive and follows a circadian pattern. *CDF1*, CDF2 and *CDF3* genes have been reported to show circadian clock-regulated expression^4^ in both tomato and Arabidopsis. Consequently, the expression patterns of these three genes *SlCDF1*, *SlCDF2 and SlCDF3* were assessed in leaves of *TDDF1* OE and WT lines under L/D condition. Oscillations in transcript of *SlCDF1*, *SlCDF2 and SlCDF3* was noticed, with an increase in transcriptional levels early in the light phase under L/D conditions in *TDDF1* OE compare with WT plants (Fig. 1e,f).

### Phenotyping of *TDDF1* OE plants under different photoperiods

**Figure 2:**
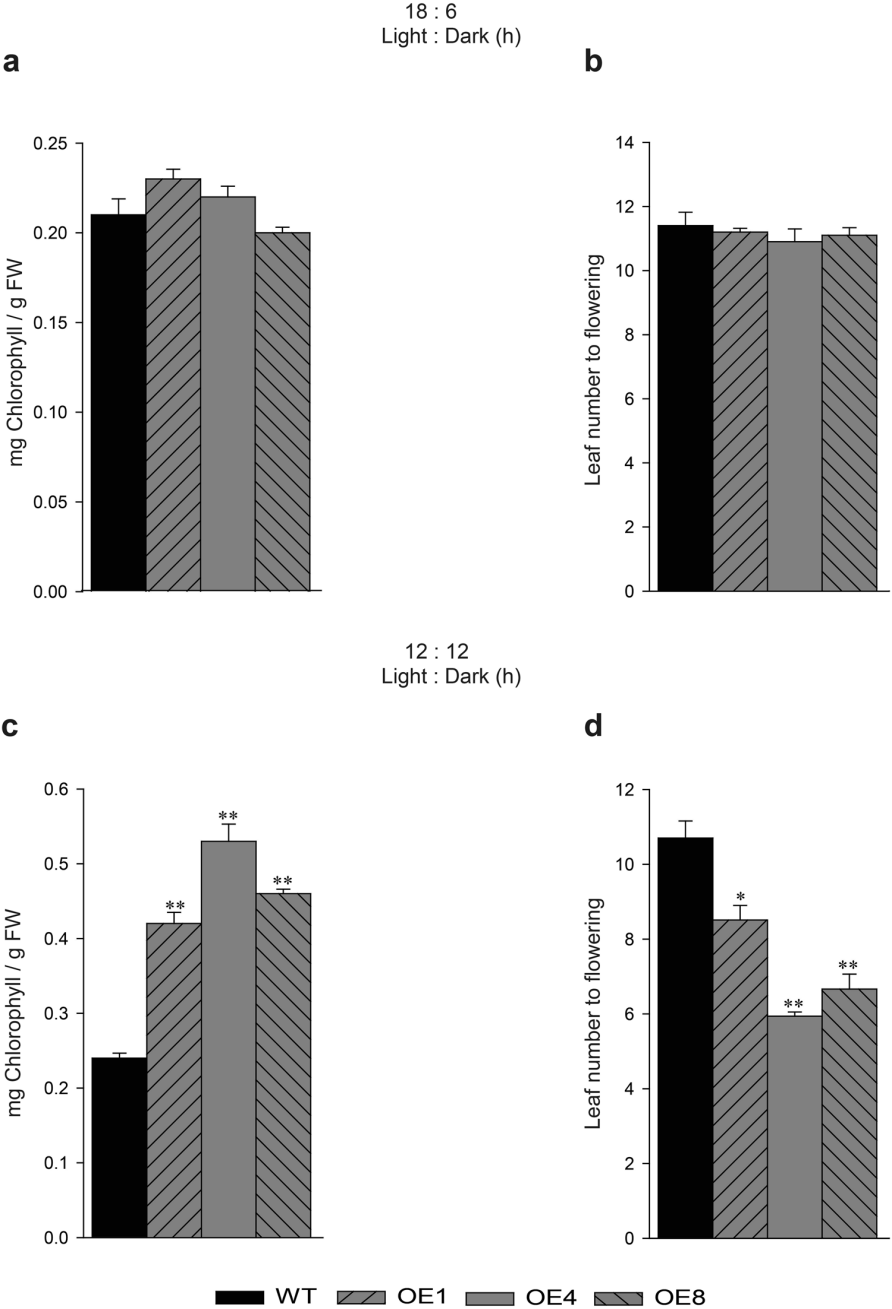
Differences in circadian phase affect the Phenotyping of *TDDF1* OE lines and WT plants. Relative chlorophyll content of *TDDF1* OE lines and WT plants grown (**a**) under neutral days (12 h light/12 h dark) and (**c**) long days (18 h light/6 h dark); leaf number to flowering of *TDDF1* OE lines and WT plants grown under (**b**) neutral days and (**d**) long days. The data shown are the mean ± SE (n = 16). Single (*P < 0.05) and double (**P < 0.01) asterisks denote statistically significant differences between the transgenic and wild-type lines.

**Figure 3:**
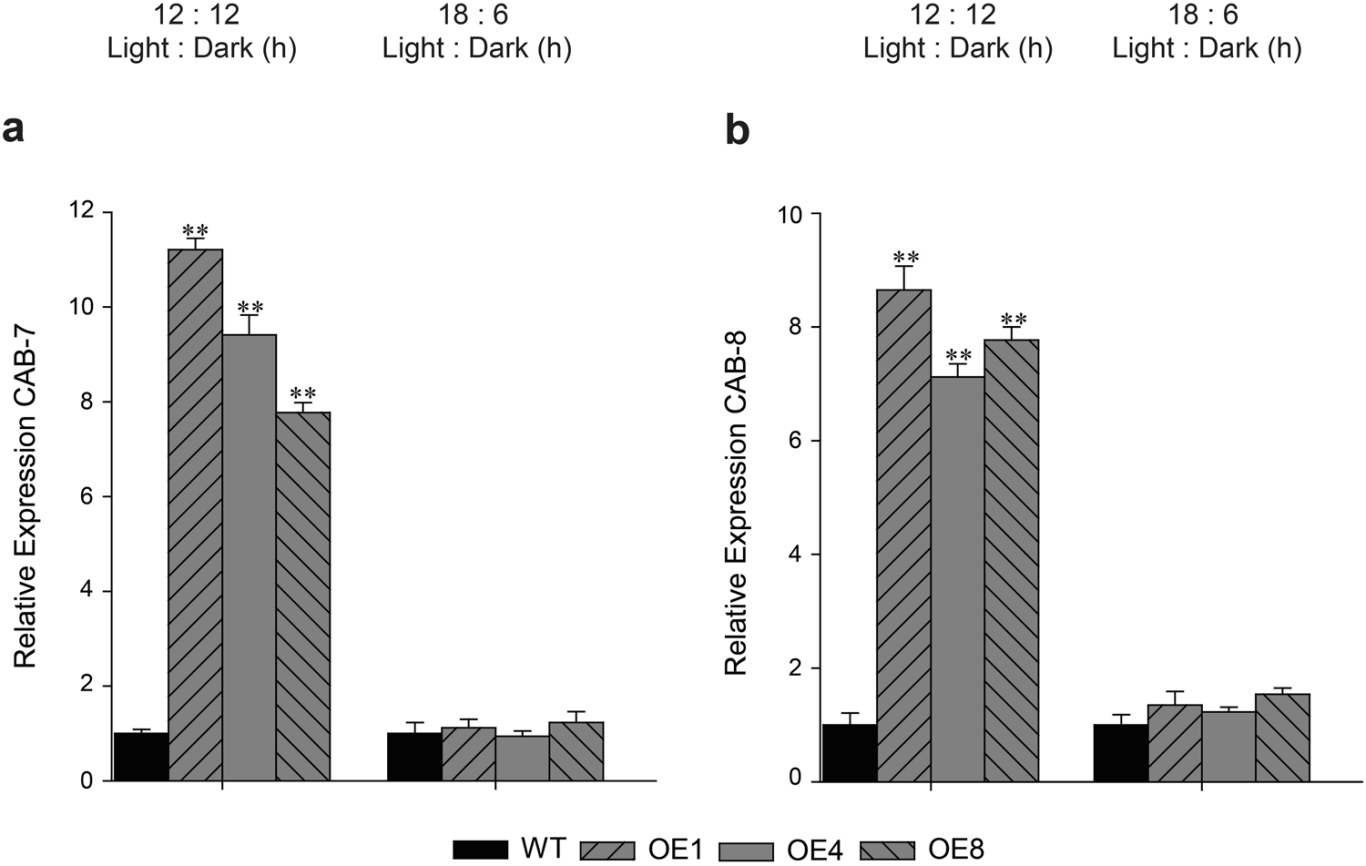
Relative expression of the two light-regulated genes, chlorophyll a/b-binding proteins (**a**) *CAB*-7 and (**b**) *CAB*-8 via qRT-PCR in *TDDF1* OE lines and WT plants under neutral days (12 h light/12 h dark) and long days (18 h light/6 h dark).

**Figure 4:**
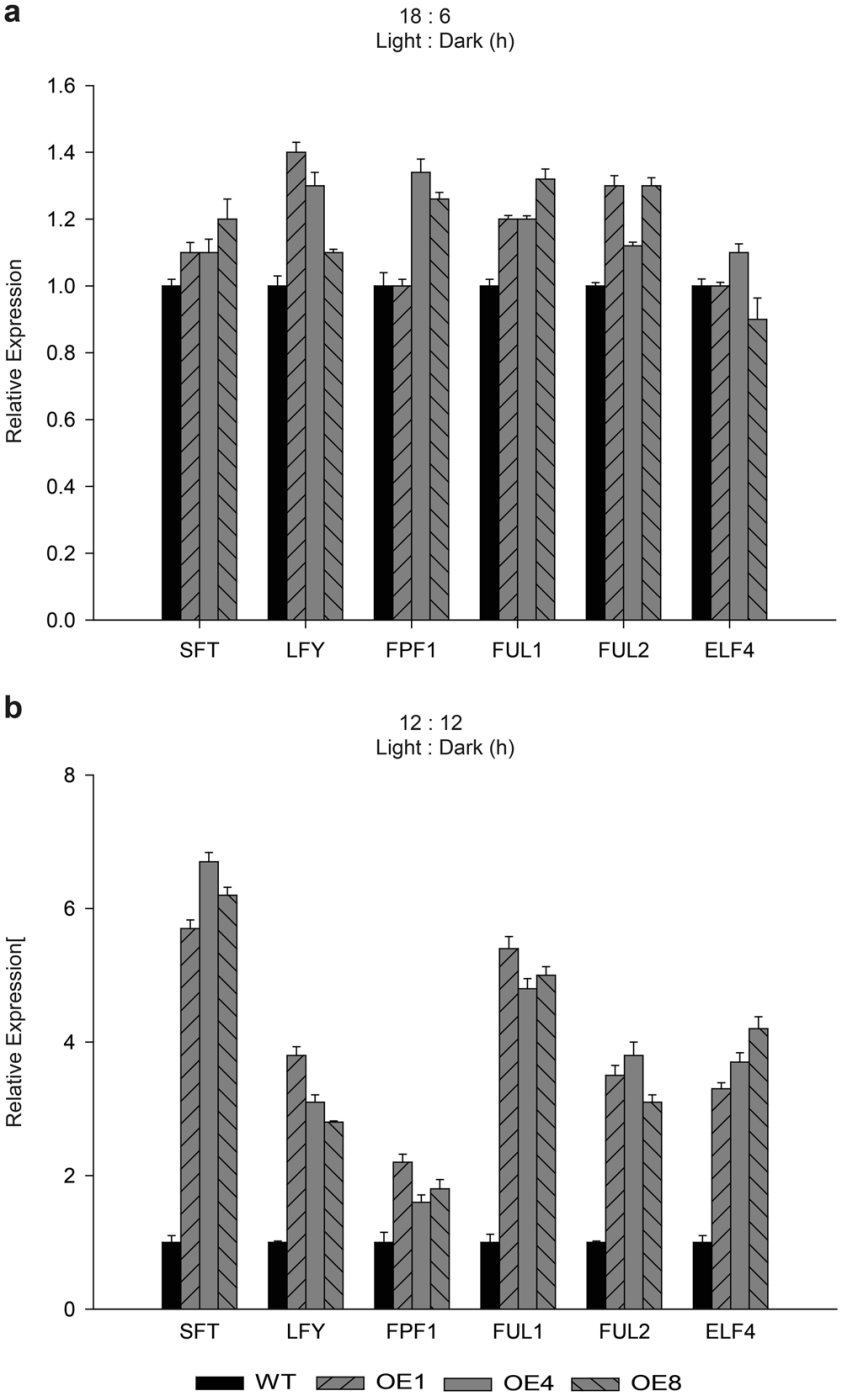
*TDDF1* overexpression accelerates early flowering. Relative expression of *SFT*, *LFY*, *FPF1*, *FUL1*, *FUL2* and *ELF4* which are known as a flowering time control genes in tomato via qRT-PCR in *TDDF1* OE lines and WT plants under (**a**) neutral days (12 h light/12 h dark) and (**b**) long days (18 h light/6 h dark). The data shown are the mean ± SE (n = 3).

**Figure 5:**
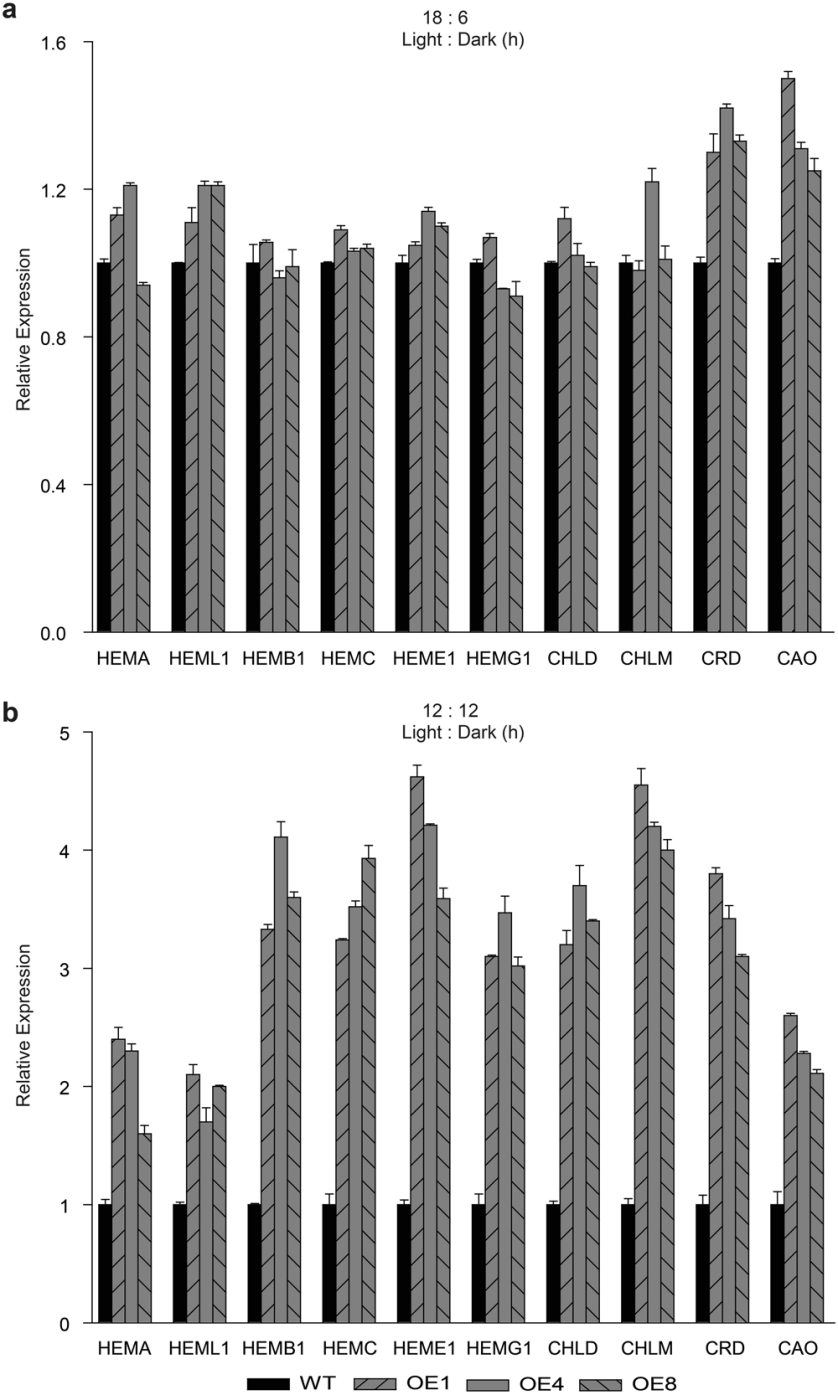
*TDDF1* overexpression enhances chlorophyll a/b biosynthesis. Relative expression of *HEMA*, *HEML1*, *HEMB1*, *HEMC*, *HEME1*, *HEMG1*, *CHLD*, *CHLM*, *CRD* and *CAO*, the key genes in chlorophyll biosynthesis pathway in tomato, via qRT-PCR in *TDDF1* OE lines and WT plants under (**a**) neutral days (12 h light/12 h dark) and (**b**) long days (18 h light/6 h dark). The data shown are the mean ± SE (n = 3).

To divulge whether overexpressing *TDDF1* affecting agro-traits under different photoperiods, leaf number to flowering and chlorophyll content was measured in WT and transgenic lines. Under long photoperiods (18/6), no significant differences were obtained in chlorophyll content and flowering time between *TDDF1* OE and WT lines (Fig. 2a,b). While, under neutral days (12/12), *TDDF1* OE lines had high chlorophyll content (up to 0.53 ± 0.023) compared with those in WT (0.24 ± 0.006) and flowered earlier than those of WT plants (Fig. 2d). The *TDDF1* OE lines formed 7.3 leaves on average before the first inflorescence, compared to 10.7 leaves of the WT plants (Fig. 2c). However, both transgenic and WT lines grown under neutral days flowered earlier than those grown under long photoperiods (Fig. 2b, d). It can be concluded that *TDDF1*, a circadian clock gene, accelerate flowering and enhance chlorophyll content under day neutral conditions, which may in turn enhance overall crop performance. For more elucidation of these results, the expression of key and regulatory genes for light-responsiveness, chlorophyll synthesis and tomato flowering were assessed in the leaves of *TDDF1* OE lines and WT plants under cycling conditions. Expression analysis of the two light-regulated genes, chlorophyll a/b-binding proteins (*CAB*-7 and 8) were measured. The results revealed significant increase in transcript levels of *CAB*-7 and *CAB*-8 genes under neutral days (12/12) in *TDDF1* OE up-to 11 and 9–folds, respectively higher than WT plants and no significant differences was observed between *TDDF1* OE and WT lines for both genes under long photoperiods (18/6) (Fig. 3a,b). In addition, function category of the flowering time control genes *SFT*, *LFY*, *FPF1*, *FUL1*, *FUL2* and *ELF4* in transgenic and wild type plants was also over-represented under cycling conditions. Under neutral days (12/12), *SFT*, *LFY*, *FPF1*, *FUL1*, *FUL2* and *ELF4* genes were strongly expressed in *TDDF1* OE lines compared to WT plants, while slightly increased in their transcript levels under long photoperiods (18/6) in *TDDF1* OE lines as compared with WT plants (Fig. 4a,b). To further understand how overexpression of *TDDF1* increases the chlorophyll content in tomato plants, we measured the transcript levels of chlorophyll biosynthetic genes *HEMA*, *HEML1*, *HEMB1*, *HEMC*, *HEME1*, *HEMG1*, *CHLD*, *CHLM*, *CRD* and *CAO* in wild type and *TDDF1*-overexpression plants under cycling conditions. Under long photoperiod (18/6), only the transcript levels of *CRD* and *CAO* were slightly increased in *TDDF1* OE lines compared to WT plants, while no significant increase was found in the expression levels of *HEMA*, *HEML1*, *HEMB1*, *HEMC*, *HEME1*, *HEMG1*, *CHLD* and *CHLM* between *TDDF1* OE and WT plants (Fig. 5a). Conversely, the transcript levels of *HEMA*, *HEML1*, *HEMB1*, *HEMC*, *HEME1*, *HEMG1*, *CHLD*, *CHLM*, *CRD* and *CAO* were strongly up-regulated in *TDDF1* OE lines compared with WT plants under neutral days (12:12) (Fig. 5b).

### Interaction between *TDDF1* and *SFT*

**Figure 6:**
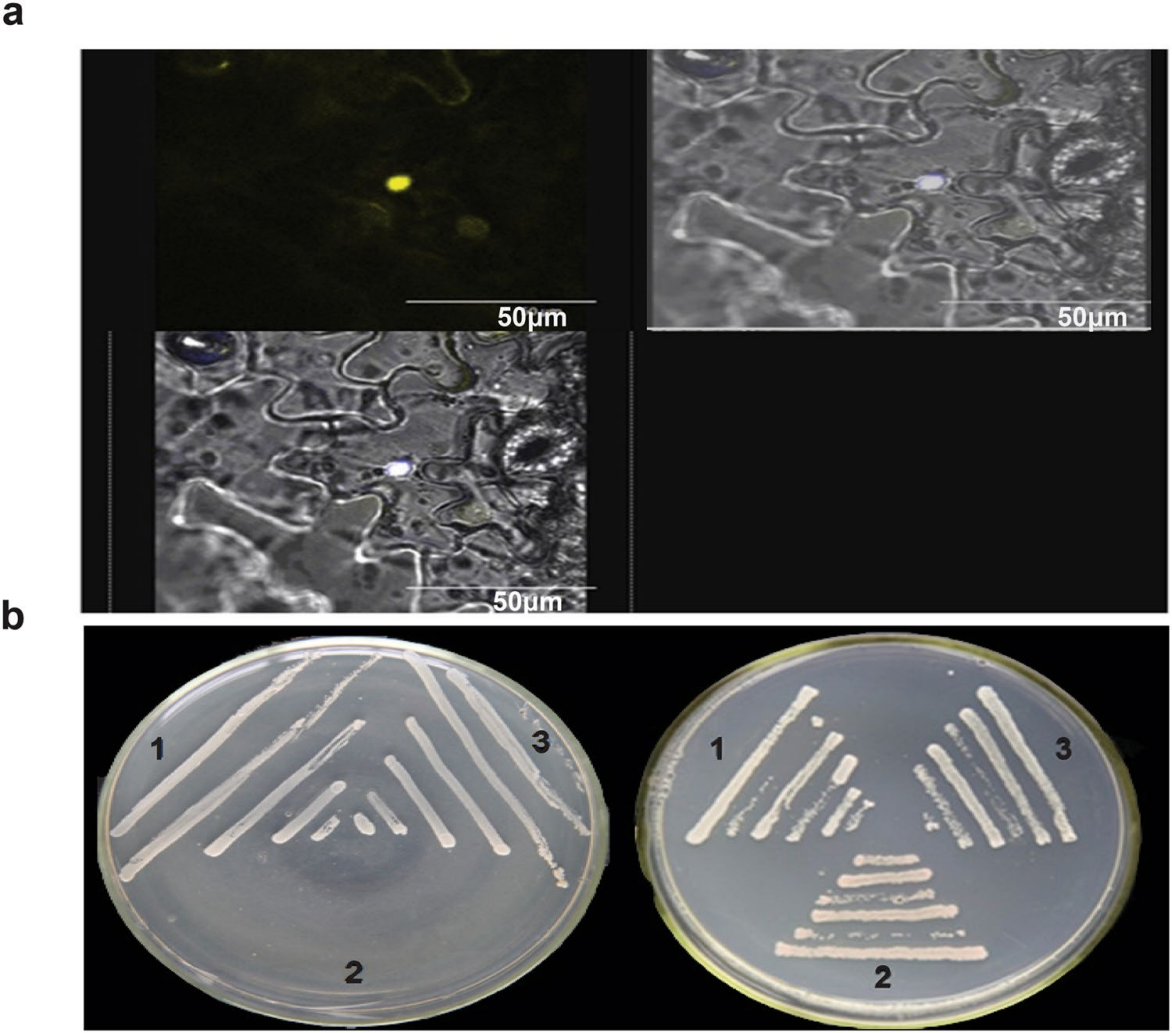
*TDDF1* interacts with *SFT in vivo*. Interaction between *TDDF1* and *SFT* in BiFC assays. (**a**) YFP fluorescence was detected only when pUCSPYNE-*TDDF1* was coexpressed with pUCSPYCE-*SFT*; (**b**) Interaction of *TDDF1* and *SFT* in transformed AH109 cells grown on SD/-Leu/-Trp (right) and SD/-Ade/- His/-Leu/-Trp (left). 1, pGBKT7−53 + pGADT7-RecT (positive control); 2, pGBKT7- Lam + pGADT7-RecT (negative control); 3, pGBKT7- *TDDF1* + pGADT7-*SFT*.

To examine whether *SFT* may be a direct target of *TDDF1*, we used a yeast one-hybrid assay. The cotransformants could not grow on the SD/-Leu/AbA 50 ng/ml medium, whereas the transformed positive control exhibited normal growth. This result suggesting that *TDDF1* may not be regulated by promoter binding with SFT. Subsequently, biomolecular fluorescence complementation (BiFC) assay were used to determine whether there is protein-protein interaction between *SFT* and *TDDF1*. Tobacco BY2 leaves (*N*. *tabacum* cv. Bright Yellow 2) were transformed with sets of pUC-SPYNE-*TDDF1*/pUC-SPYCE-*SFT* and pUC-SPYNE-*TDDF1*/pUC-SPYCE (control). We observed strong YFP fluorescence when pUC-SPYNE-*TDDF1* was coexpressed with pUC-SPYCE-*SFT*, while no signal was observed in the control leaves (Fig. 6a). This result indicated that *TDDF1* interacts with *SFT*. To further confirm the interaction between *TDDF1* and *SFT*, we performed yeast two-hybrid assay. Two different transformants harboring pGBKT7-*TDDF1* and pGADT7-*SFT* were able to grow on SD/-Ade/-His/-Leu/-Trp medium, whereas the positive and negative controls showed the expected results (Fig. 6b). The results of BiFC and Yeast two hybrid demonstrated that *TDDF1* can physically interact with *SFT*.

### *TDDF1* expression was induced by various stress conditions and hormones

The expression patterns of *TDDF1* were investigated under different stress and hormone treatments. The *TDDF1* gene was significantly induced by some of the stress conditions. Under drought stress, the *TDDF1* transcripts initially accumulated to 16-fold of the control (non-stressed plants) after 6 h and then decreased after 12 h. Under high salt stress (200 mM NaCl), *TDDF1* expression increased after 1, 3, 6, and 10 h, approximately up to 2, 3, 4 and 6-folds, respectively. Wounding resulted in a quick increase within 1 m, reaching maximum after 3 m and then declined. Interestingly, subjecting tomato plants to cold stress (4 °C) resulted in the accumulation of *TDDF1* transcripts up to 16-fold after 6 h. On the other hand, heat stress (40 °C) gradually up-regulated the expression by approximately 4-fold after 6 h and stabilized it after 12 h. Regarding phytohormone treatments, the *TDDF1* transcripts rapidly increased up to 26-fold within 12 h in response to ABA (100 μM). Almost same trend was noticed for transcript induction by SA (100 μM) treatment, which caused 20-fold increase in *TDDF1* mRNA accumulation. ETH (100 μM), caused gradual enhancement in transcript level of *TDDF1* up to 4-fold. In contrast to other phytohormones, GA (100 μM) decreased the endogenous mRNA level of *TDDF1* (Fig. S4). Overall, the ABA, SA and drought stresses exerted the strongest effects over all the hormones or stressors.

### *TDDF1* positively regulates abiotic and biotic stress resistance in tomato

To evaluate the effects of changes in expression of *TDDF1* in response to abiotic stresses, drought and salt stress tolerance tests were performed. WT and *TDDF1* OE plants treated *in vitro* with mannitol (200 mM), NaCl (100 mM) and ABA (3 µM) in MS medium at seedling stage. Results showed that *TDDF1* OE seedlings were more tolerant to mannitol and salt stress than WT seedlings (Fig. S5a,b). Conversely, ABA treatment decreased the seedling length and weight of *TDDF1* OE lines by 27% and 31%, respectively, while WT seedlings decreased only by 12% (Fig. S5c).

**Figure 7:**
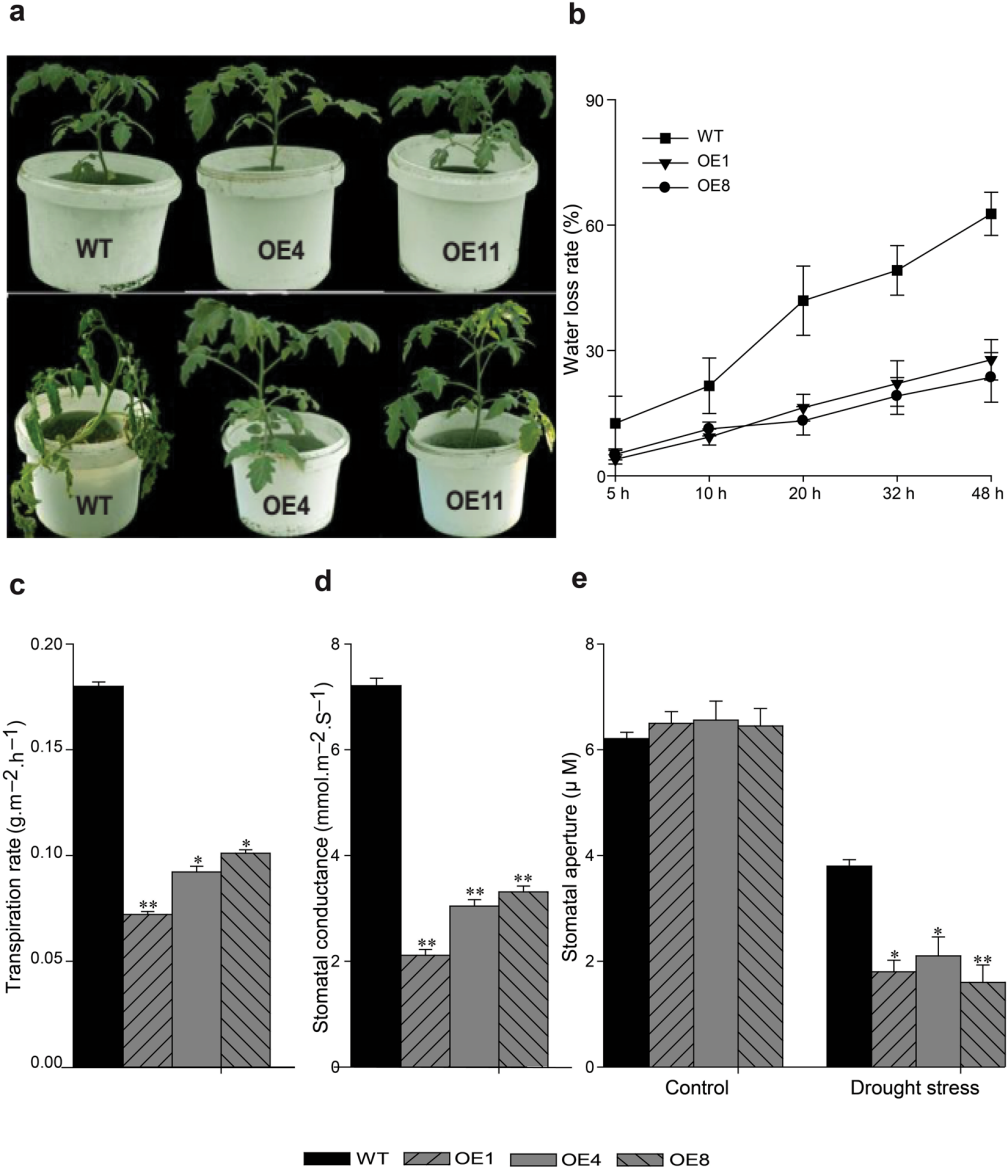
*TDDF1* expression empowers resistance towards abiotic stress in tomato. (**a**) Drought tolerance assay for *TDDF1* OE and WT plants; (**b**) Water loss assay for the detached leaves excised from *TDDF1* OE and WT plants. The detached leaves were collected from mature plants at the same position and placed on a Wattman paper at room temperature. The mean weight was measured at five time points (n = 6) leaves from wild types and transgenic plants; (**c**) Transpiration rate; and (**d**) stomatal conductance of the detached leaves; The two parameters were detected after the detached leaves were placed on a filter paper and exposed under white florescent light for 5 h; (**e**) Stomatal aperture of wild type and transgenic plants under drought stress. Leaves were detached from 2-month old wild type and transgenic plants and subjected to drought stress for 5 h *in vitro*. The lower surfaces of the leaves with or without stress were examined under a microscope. Single (*P < 0.05) and double (**P < 0.01) asterisks denote statistically significant differences between the transgenic and wild-type lines under drought stress.

The *TDDF1* OE and WT plants were challenged with drought stress by suspending watering to one-month-old plants. After two weeks without watering, WT plants started to wilt. One week later, WT plants showed more severe wilting symptoms. However, all the *TDDF1* OE plants remained turgid and showed substantially delayed wilting over twenty days of drought. *TDDF1* OE plants recovered efficiently three days after rewatering but WT plants failed to recover (Fig. 7a). The idea that *TDDF1* positively influences drought tolerance was further supported by *in vitro* water loss assays (Fig. 7b). *In vitro* drought tests revealed that transpiration rate and stomatal conductance of the *TDDF1* OE plants was significantly reduced compared with WT plants (Fig. 7c,d). Furthermore, the stomatal aperture of *TDDF1* OE plants was significantly smaller than that of WT plants under drought stress (Fig. 7e). These results confirmed the notion that *TDDF1* functions in drought stress by affecting stomatal size and regulating stomatal movements to reduce water loss.

**Figure 8:**
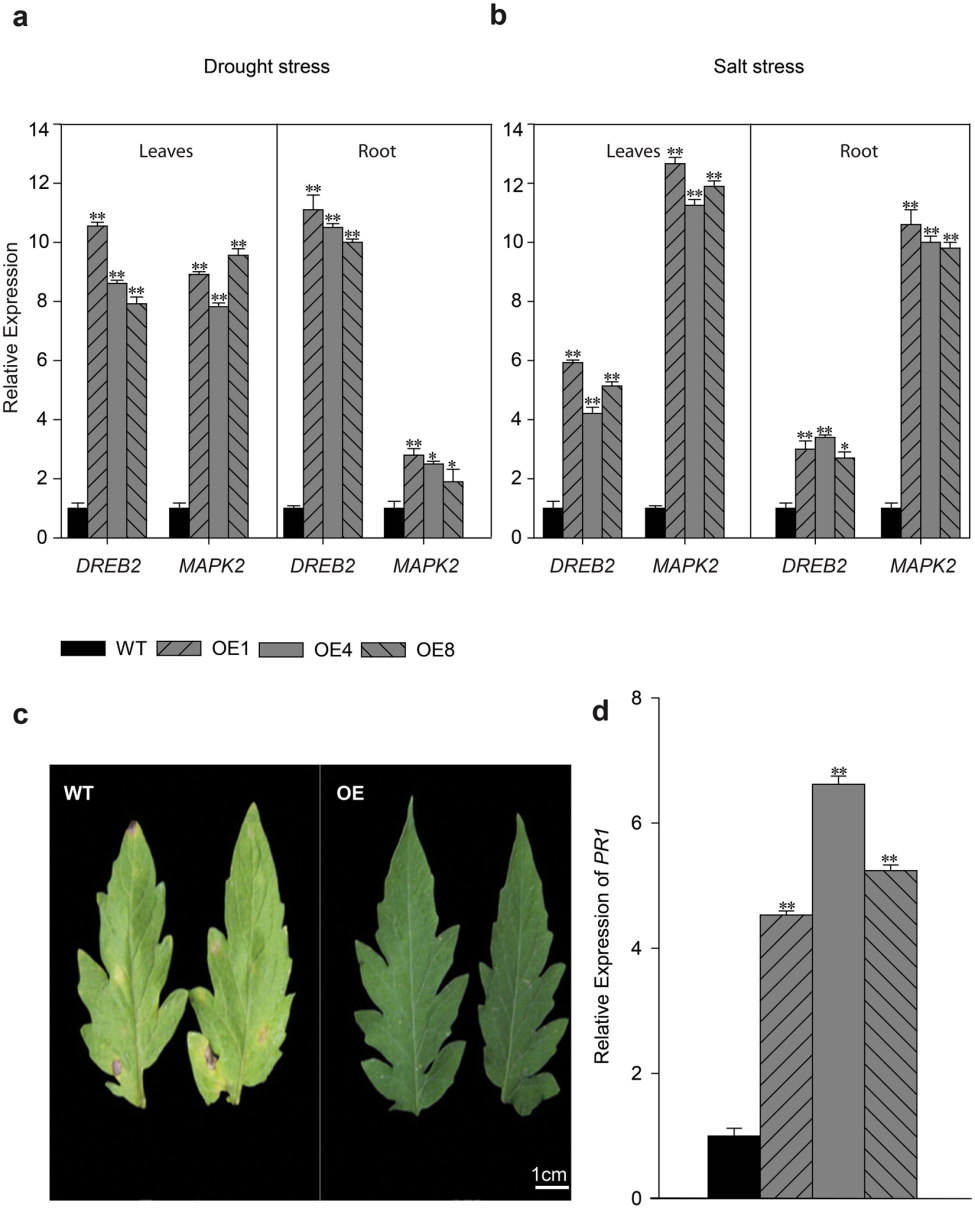
*TDDF1* overexpression enhances abiotic and biotic resistance in tomato. Relative expression levels of (**a**) *DREB2* and (**b**) *MAPK2*, the key genes related to drought tolerance in WT and *TDDF1* OE lines. (**c**) The lesion measurement in diseased leaves of WT and transgenic plants five days post inoculation with *Phytophthora infestans*. Ten leaves from each individual line were inoculated; (**d**) Relative expression of *PR1*, a key gene for pathogen resistance, in WT and *TDDF1* OE lines via qRT-PCR. The data shown are the mean ± SE (*n* = 3). Single (*P < 0.05) and double (**P < 0.01) asterisks denote statistically significant differences between the transgenic and wild-type lines.

To further confirm the idea that OE *TDDF1* result in drought and salt stress tolerance, transcript levels of *DREB2* and *MAPK2* that are key genes of abiotic stress were analyzed both in *TDDF1* OE and WT plants. The results showed that *DREB2* and *MAPK2* genes were significantly up-regulated in the leaves and root of *TDDF1* OE lines compared with WT plants under both drought and salt conditions (Fig. 8a,b). *Phytophthora infestans* is one of the most devastating pathogens of tomato, which causes late blight disease. Resistance to late blight conferred by *TDDF1* was tested by examining plants inoculated with *Phytophthora infestans*. *TDDF1* OE showed a slow progress of disease when compared to WT plants (Fig. 8c). For further confirmation, we measured the relative expression of *Pathogenesis Related Protein1* (*PR1*), which is known as resistance marker gene induce in response to pathogen and herbivores attack. The relative expression of *PR1* was approximately between 4.5 to 6.6-fold higher in *TDDF1* OE lines than WT plants (Fig. 8d).

### Hormonal balance under water stress and high salinity in *TDDF1* OE and wild-type tomato

The endogenous JA, ABA, SA and GA contents of 2-months-old *TDDF1* OE and WT plants under normal (no stress) or without watering conditions for 7 days and 48 hours after exposure to 200 mM NaCl were determined as indicators of the hormonal status in leaves. No significant differences were found between *TDDF1* and WT plants for ABA and JA concentrations under normal condition, while SA concentration was slightly higher in *TDDF1* OE lines than WT plants under normal condition. On the other hand, GA level in *TDDF1* OE lines was slightly lower than WT plants under normal condition. Water stress induced endogenous ABA, JA and SA contents, which reached in *TDDF1* OE lines approximately 2-fold higher than WT leaves. Conversely, due to water stress the GA level was decreased in *TDDF1* OE lines compared to WT plants (Fig. S6). A marked increase of endogenous ABA and JA contents was recorded in *TDDF1* OE lines in response to salt stress. In contrast to ABA and JA, GA content rapidly declined in *TDDF1* OE leaves exposed to salinity and was the most sensitive of all four phytohormones to the NaCl treatment. Similar to GA, SA level in *TDDF1* OE leaves declined significantly in response to salinity as compared to WT plants.

### *TDDF1* positively regulates the expression pattern for key genes of ABA, JA and SA pathways

**Figure 9:**
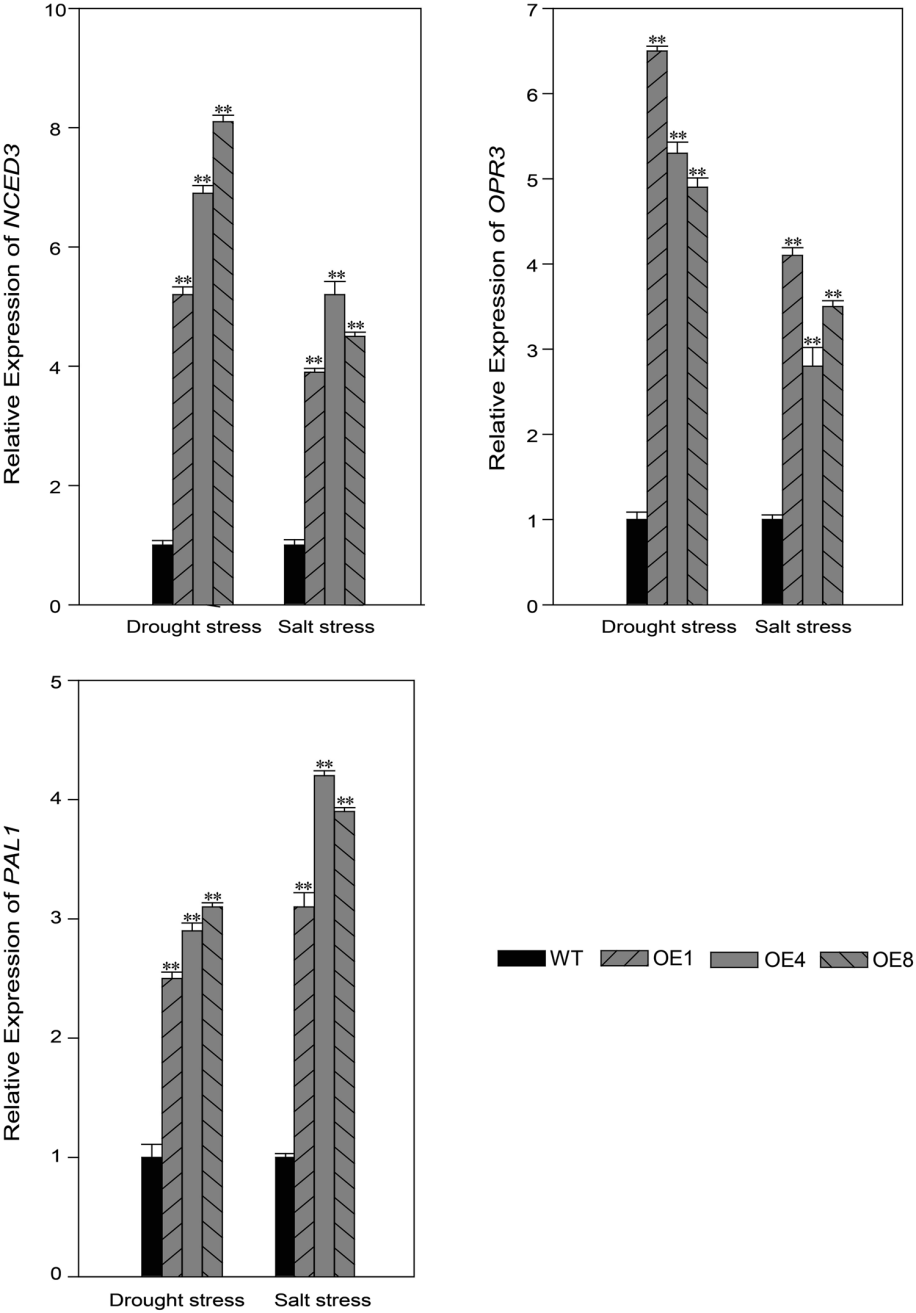
*TDDF1* positively regulates gene expression pattern for *NCED3*, *OPR3* and *PAL1*, the key genes of ABA, JA and SA biosynthesis pathway. Single (*P < 0.05) and double (**P < 0.01) asterisks denote statistically significant differences between the transgenic and wild-type lines.

The results of hormonal analysis showed significant increase in ABA, JA and SA levels under drought and salinity stress due to overexpression of *TDDF1*. To determine whether overexpressing *TDDF1* could also affect the expression of key genes for the biosynthesis of these hormones, gene expression of *NCED3*, *OPR3* and *PAL1*, which are known as key genes in ABA, JA and SA biosynthesis pathways, respectively. These genes were assessed in the leaves of *TDDF1* OE lines and WT plants under drought and salinity stress. Water deprivation strongly induced expression of *NCED3*, *OPR3* and slightly that of *PAL1* in *TDDF1* OE lines in comparison with WT plants. In the same order, the expression level of these three genes was enhanced in *TDDF1* OE lines than in WT plants under salinity stress (Fig. 9).

### *TDDF1* improves photosynthesis and alleviates oxidative stress during water deficit conditions

Drought and salt stresses are known determinants that promote substantial physiological and metabolic rearrangements in plants^39, 40^. Therefore, we carried out a targeted metabolite profiling and chlorophyll analysis under cycling conditions to address the question of whether the ectopic expression of *TDDF1* in tomato translates into a detectable alteration of the plant’s metabolome under water stress condition or not? The chlorophyll (A and B) content and biomass of the *TDDF1* OE lines grown under optimal (at 100% FC) and reduced watering regimes (at 25% FC) were measured. Under long photoperiod (18/6), no significant differences were found between *TDDF1* OE lines and WT plants for chlorophyll (B) content under the optimal watering regime, but chlorophyll (A) slightly increased in *TDDF1* OE compared to WT plants. However, chlorophyll (A and B) contents under water stress were higher in *TDDF1* OE lines than WT plants during long photoperiod (Fig. S7a). Conversely, under neutral days (12:12) significant differences in the chlorophyll (A and B) were observed between *TDDF1* OE and WT plants in both normal and water stress conditions (Fig. S7b). In addition, no significant differences were found between *TDDF1* OE lines and WT plants for biomass content under the optimal watering regime (Fig. S8a). However, reduced watering for one month severely reduced the biomass of WT plants (approximately 54%), whereas that of *TDDF1* OE plants decreased only about 23.5% as compared to control (Fig. S8a). These results clearly indicate the role of *TDDF1* towards abiotic stress. For further confirmation, proline, soluble sugars and MDA, known as an important indicator biomolecules of plant oxidative stresses, were measured under normal and water stress conditions. Under water stress the proline concentrations in OE lines were 3–4 folds higher than that in WT plants (Fig. S8b). Consistent with proline results, a marked increase of soluble sugars content under drought stress were recorded in *TDDF1* OE lines compared to WT plants (Fig. S8d). In contrast, MDA significantly decreased in *TDDF1* OE lines as compared to that in WT plants under water stress (Fig. S8c). Hence, *TDDF1* can positively improve drought stress tolerance. Oxidative stress refers to elevated intracellular levels of reactive oxygen species (ROS) that cause damage to lipids, proteins and DNA^41^. To determine whether overexpressing *TDDF1* affected ROS production, leaves of *TDDF1* OE and WT lines were detached. After 5 hours of drought *in vitro*, wilt symptoms were more obvious in leaves of WT than that of *TDDF1* OE leaves. DAB staining showed that WT plants accumulated more hydrogen peroxide coupled with deeper brown spots than those in *TDDF1* OE lines under drought stress (Fig. S9). Therefore, *TDDF1* can alleviate oxidative damage caused by excessive production of ROS and thus improve oxidative stress tolerance.

## Discussion

Plants being sessile are subjected to various environmental changes and stresses including day length, drought, salinity and various pathogens during their life cycles that may adversely affect their productivity alongwith the transition and maintenance of flowering. The final yield will always be less under stress condition and different photoperiods because being a biological entity plants cannot perform all the biological and metabolic processes efficiently. The ability of crop to confer multiple desirable traits in a single variety is highly demanded in this advance era of molecular biology. This idea usually requires selection of multiple alleles of more than one gene^18^ for broad spectrum resistance against several adverse environmental conditions and biological hurdles. Consequently, pyramiding all desirable genes by single breeding program or even genetic engineering is a cumbersome task and consuming a lot of resources. Therefore, plant scientist needs to address the bottlenecks of gene identification and role of different genes at the same time. Our results confirm the expression of *TDDF1* and identification of *Sp*DOF17 as tomato Dof gene. We validate the expression pattern of *TDDF1* in response to abiotic stress conditions and different photoperiods.

Transcription factors are a group of major proteins which regulate gene expression through binding to *cis*-elements in the promoter regions upstream or downstream of target genes. As target gene regulators, transcription factors are involved in myriad of biological processes, such as growth, development, cell cycle, metabolism, circadian control, light response and biotic/abiotic stress tolerance. Increasing evidence suggests that these stresses are modulated by multiple hormone signaling pathways regulated by abscisic acid (ABA), jasmonic acid (JA), ethylene (ET), salicylic acid (SA), as well as reactive oxygen species (ROS)^23, 24^.

Interestingly, *TDDF1* expression changed with the circadian rhythm and coincided with stomatal movement in 24 hour duration cycle. A *cis*-acting regulatory element involved in the circadian control (circadian CAACAGCATC) and some light responsive elements (CATT-motif, AE-box, ATCT-motif, BOX I, GAG-motif, I-box, GAG-motif and TCT-motif) were found in the *TDDF1* promoter region. *TDDF1* lies phylogenetically most close to *OsRdd1* ^35^, *SlCDF1*, *SlCDF2* and *SlCDF3* ^5^ (Fig. S1), which have been reported to show circadian clock-regulated expression^4^. Under L/D conditions, *SlCDF1*, *SlCDF2* and *SlCDF3* showed daily oscillations, with an increase in transcriptional levels early in the light phase in *TDDF1* OE compared with WT plants. Furthermore *TDDF1* overexpression accelerates the flowering time consistent with previous report, which showed that overexpressing *OsRdd1* exhibited early flowering^35^. In addition, the expression levels of *SFT*, *LFY*, *FPF1*, *FUL1*, *FUL2* and *ELF4* were activated in *TDDF1* OE lines compared to wild-type plant. Hence, *TDDF1* may associate with the expression of flowering time control genes. In the same manner, *TDDF1* overexpression enhanced the chlorophyll (A and B) synthesis by up-regulating the expression levels of biosynthetic genes *HEMA*, *HEML1*, *HEMB1*, *HEMC*, *HEME1*, *HEMG1*, *CHLD*, *CHLM*, *CRD* and *CAO* in tomato.

*TDDF1* expression was also regulated in response to abiotic and biotic stress conditions and several hormones, which may be due to the presence of drought, salt, heat and MeJA stress responsive *cis*-elements (CGTCA-motif, TGACG-motif, MBS, MYB, RAV and TC-rich) in the promoter region of *TDDF1*. These *cis*-elements are the core elements for stress response^42^. Presence of these *cis*-elements alongwith transcript change in response to drought, salt, wounding, cold and heat shock treatments indicate that *TDDF1* can bind to abiotic stress resistance genes. The increase in expression levels of *DREB* and *MAPK2* in *TDDF1* OE lines indicates that *TDDF1* is involved in abiotic stress resistance in tomato through up-regulating abiotic stress responsive genes.

The analysis of *TDDF1* promoter region also revealed some biotic stress-response elements such as CGTCA-motif, TGACG-motif and TC-rich elements. *Pathogenesis Related Protein1* (*PR1*), which is known as resistance/defense marker gene, was up-regulated in *TDDF1* OE plants showing elevated resistance to late blight, which further illustrates the involvement of *TDDF1* in biotic stress resistance.

Drought is sort of oxidative stress in plants, while proline, soluble sugars and MDA are osmoprotectants and known as an important indicator of plant oxidative stresses^43^. The present study showed that the proline content in *TDDF1* OE lines was higher than that in WT plants under drought stress (Fig. S8). This increase in proline and soluble sugar amounts enhanced drought tolerance in *TDDF1* OE plants. This result is in consistence with corresponding tolerance reported earlier in tomato, tobacco and Arabidopsis^43–45^. In addition, *TDDF1* was also significantly induced by paraquat, an inducer of ROS. Under drought and paraquat stress conditions, the results of DAB staining assay indicated that *TDDF1* plays an important role in alleviating oxidative stress in tomato.

### How does *TDDF1* work as flowering accelerator and protector against various stresses?

The mechanism of the *TDDF1:* When tomato plants exposed to the abiotic stresses such as drought and salt, the ABA content significantly increased in *TDDF1* OE plants. Drought and salt tolerance mediated by elevated ABA may be attributed to the reduction in stomatal aperture of *TDDF1* OE plants which is likely to be the major factors contributing to the drought and salt tolerance of *TDDF1*. Under drought and salt stress, the higher expression of *NCED3* (a key gene in ABA biosynthesis) in *TDDF1* OE lines, led to stomatal movement^46^ and further maintains normal photosynthesis in *TDDF1* OE plants. In the same manner, resistance to late blight conferred by *TDDF1* may be due to the increase in JA and SA levels which in turn, induce the expression of many stress-related genes specifically PR genes. Overexpression of *TDDF1* activates some stress responsive genes such as *DREB2*, *MAPK2* and *PR1*, which led to the accumulation of proline and soluble sugars to alleviate oxidative stress by eliminating ROS production. Furthermore, the *TDDF1* transcript exhibited large daily oscillations irrespective of light conditions, indicating that *TDDF1* expression was regulated by the circadian clock. *TDDF1* regulating the transition of flowering, might be due to the activation of /and protein-protein interaction with *SFT*, a floral inducer gene in tomato. Altogether, these various mechanisms result in enhanced stress resistance and circadian-controlling early flowering in tomato *TDDF1* OE lines.

## Materials and Methods

### Plant material and Bioinformatics analysis

The tomato *TDDF1* gene refers to *Solanum pennellii Dof17* as annotated in the tomato genome sequence. Tomato *S*. *lycopersicum* variety Ailsa Craig (AC) was used as wild type. Sequencing results of *TDDF1* were used to search for homology, based on protein and nucleotide sequences, at NCBI (http://www.ncbi.nlm.nih.gov) and SOL genomics network (http://www.solgenomics.net). The nucleotide and deduced amino acid alignments were computed by the ClustalW program employing standard parameters, and shaded in GENEDOC. The phylogenetic tree was generated in MEGA 5 software, using the Neighbour joining method. The cis-acting regulatory elements in the promoter region were analyzed using the PlantCARE^47^ and PLACE databases^48^.

### Plant growth and stress treatments

For gene expression profiling analysis, 2-month old uniformly developed tomato plants (LA716, S. pennellii) were grown in a greenhouse under a 14/10 h light/dark regime at 25 °C and subjected to various stresses or hormone treatments. Salt stress was induced by watering the plants with 200 mM NaCl solution. Drought stress was simulated by placing detached leaves on a filter paper in 70% relative humidity at 25 °C. Cold or hot stress conditions were imposed by transferring the plants to a growth chamber and holding the plants at 4 °C or 40 °C, respectively. Wounding was performed by pinching the leaves with forceps. For hormone treatments and oxidative stress, 3 and 100 μM ABA, 100 μM ethylene (ETH), 100 μM gibberellic acid (GA3), 100 μM SA, and 100 μM paraquat were directly sprayed onto tomato plants. After each treatment, leaves from different plants (three biological replicates) were collected and immediately frozen in liquid nitrogen and stored at −80 °C until RNA isolation.

### Vector construction and plant transformation

*TDDF1* was amplified from the cDNA and genomic DNA of *S*. *pennellii*, using specific primers based on the unigene sequence, SGN-U569211 (Table S1). The amplified PCR product was cloned into the pMD18-T vector, and the correct sequence was confirmed by sequencing. The pMD18-T vector containing the *TDDF1* cDNA was double digested using *Xba*I and *Kpn*I and ligated into the plant binary vector pBI121 under the control of a strong constitutive promoter (CaMV35S), cleaved with *Xho*I and *Sac*I. *TDDF1* construct was transformed into tomato *S*. *lycopersicum* variety Ailsa Craig (AC), mediated by *Agrobacterium tumefaciens* strain C58. Empty vectors were transformed as controls. To investigate the subcellular localization of *TDDF1*, we bombarded the construct (35 S::*TDDF1*:EGFP) into tobacco (*N*. *tabacum*) leaves.

### Transgenic Analysis

The T_0_ and T_1_ plants were selfed, the selected T2 and T3 seeds transgenic lines were germinated on kanamycin (100 mg l^−1^) selection medium. Kanamycin-resistant plants were further confirmed through PCR using 35S-F (a forward primer in the 35S promoter) and TDDF1-R (a reverse primer specific for *TDDF1*) (Table S1). The kanamyicn spraying test was used in the genetic segregation analysis^49^ and three T_2_ OE lines (OE1, OE4 and OE8) were used for further study.

### Expression Analyses

Total RNA was extracted with Trizol reagent (Invitrogen) and the first-strand cDNA was synthesized using 3 μg of RNA and 200 U of M-MLV reverse transcriptase (Invitrogen) according to the manufacturer’s protocol. RT-PCR was carried out to amplify a 400 bp fragment of *TDDF1* with 31 cycles using the first-strand cDNA as a template. In addition, actin was amplified with 24 cycles as an internal control. Real-time PCR was performed on an optical 96-well plate in an AB StepOnePlus PCR system (Applied Biosystems) by using SYBR Premix Reagent F-415 (Thermo Scientific). Relative gene expression was calculated using a relative quantification method^50^. All primers used in this analysis are listed in (Table S1).

### Yeast two-Hybrid Assay

For yeast two-hybrid analysis, two different constructs pGBKT7-*TDDF1* and pGADT7-*SFT* were created by inserting *TDDF1* and *SFT* into pGBKT7 and pGADT7 vectors separately. The two plasmids were cotransformed into *Saccharomyces cerevisiae* strain AH109. Simultaneously, we also transformed pGBKT7–53 and pGADT7-RecT as positive control, and pGBKT7-Lam and pGADT7-RecT as negative control, which were provided with the BD Matchmaker library construction and screening kits. The transformants were then tested on SD/-Leu/-Trp and SD/-Ade/-His/-Leu/-Trp mediums.

### Stress tolerance assays in the transgenic tomato plants

Three independent experiments were conducted to elucidate the function of *TDDF1* in drought tolerance. In each experiment, *TDDF1* OE and WT plants were grown in pots containing a soil mix (1:1, vermiculite: houmus). For the survival rate assay, the assay plants were kept in growth chamber and water was withheld for 22 days before re-watering. For water-loss assays, leaves were detached from *TDDF1* OE and WT plants then placed, abaxial side up, on open petri dishes. The leaves were weighed individually at different time points to determine the rate of water loss. For drought and salt treatments, *TDDF1*OE and WT seedlings (3-day old) were transferred to MS medium containing 200 mM Mannitol, 100 mM NaCl, respectively. After 15 days, both shoot and root lengths were measured.

Meanwhile, some stress-related biochemical markers were examined under drought stress at 25% and well-watered conditions at 100% field capacity (FC). The drought stress treatment was initiated at the four-leaf stage. The chlorophyll (A and B) contents were determined according to^51^. The soluble sugars, proline, and malondialdehyde (MDA) were determined using previously described methods^52, 53^. All measurements were conducted with three replicates.

To investigate the oxidative tolerance of *TDDF1*, excised 5 h leaves from well-watered transgenic and Wt lines were stained using the diaminobenzidine (DAB) method according to^54^. The oxidative tolerance of *TDDF1* was further studied by spraying 100 μM of paraquat (inducer of reactive oxygen species, ROS) onto well-watered plants for 24 h. The oxidative damage of the leaves was then determined using the above-mentioned DAB staining method.

### Pathogenesis assay with *Phytophthora infestans*

Aggressive isolates of *Phytophthora infestans* (99183) were used for inoculation. Infection was carried out on the surface of detached leaves from *TDDF1*OE and WT lines. Leaves were kept under 16-h photoperiod at 20 °C and 100% humidity. The disease symptoms were observed every two days, over a period of one week. This experiment was repeated three times under similar conditions. Lesion growth rate (LGR) was observed and calculated (in cm^2^) on the surface of leaves, using a digital vernier caliper.

### Hormone Analysis

Hormone extraction and analysis were carried out following the procedure described in with slight modifications^55^. Frozen fresh tissue (0, 2 g) was spiked with 100ng of d6-ABA, 100 ng of dihydrojasmonic acid and 100 ng of d6-SA, and homogenized with 5 ml of distilled water. After centrifugation at 4000 × g at 4 °C, supernatants were recovered and pH adjusted to 3 with 30% acetic acid. The acidified water extract was partitioned twice against 3 ml of diethyl ether. The organic upper layer was recovered and vacuum evaporated in a centrifuge concentrator (Speed Vac, Jouan, Saint Herblain Cedex, France).The dry residue was then resuspended in a 10% MeOH solution by gentle sonication. The resulting solution was passed through0.22 µm regenerated cellulose membrane syringe filters (AlbetS.A., Barcelona, Spain) and directly injected into a UPLC system (AcquitySDS, Waters Corp., Milford, MA, USA). Analyses were separated by reversed-phase (Nucleodur C18, 1.8 µm 50 × 2.0mm, Macherey- Nagel, Barcelona, España) using a linear gradient of ultrapure H2O (A) and MeOH (B) (both supplemented with 0.01% acetic acid) at a flow rate of 300µl min−1. The gradient used was: (0–2 min) 90:10 (A:B), (2–6 min) 10:90 (A:B), and (2–6–7 min) 90:10 (A:B). Hormones were quantified with a Quattro LC triple quadruple mass spectrometer (Micromass, Manchester, UK) connected online to the output of the column through an orthogonal Z-spray electrospray ion source. Quantitation of plant hormones was achieved by external calibration with standards of known amount.

### Ethical approval

For studies with animals, all institutional and national guidelines for the care and use of laboratory animals were followed.

## Electronic supplementary material

Supplementary Information
