# Supplementary material for: The Tomato DOF Daily Fluctuations 1, TDDF1 acts as flowering accelerator and protector against various stresses
Source: Sci Rep. 2017 Aug 31;7:10299. doi: 10.1038/s41598-017-10399-7 (PMC5578996; doi:10.1038/s41598-017-10399-7)
Supplement: Supplementary file 1 — Supplementary Information [file 41598_2017_10399_MOESM1_ESM.pdf]

Supplementary Data

The Tomato DOF Daily Fluctions 1, TDDF1 acts as flowering accelerator and protector against various stresses

Mohamed Ewas[1,2], Eman Khames[3], Khurram Ziaf[4], Raheel Shahzad[1], Elsayed Nishawy[1,2], Farhan Ali[5], Hizar Subthain[1], Mohamed H. Amar[2], Mohamed Ayaad[6], Omran Ghaly[2] & Jie Luo[†1]

[1] National Key Laboratory of Crop Genetic Improvement and National Center of Plant Gene Research (Wuhan), College of Life Science and Technology, Huazhong Agricultural University, Wuhan, Hubei 430070, China.
[2] Genetic Resources Department, Deserts Research Center (DRC), Cairo, Egypt.
[3] College of Pharmacy, Tanta University, Cairo, Egypt.
[4] Institute of Horticultural Sciences, University of Agricultural, Faisalabad, Pakistan.
[5] Cereal crops research Institute (CCRI) Nowshera, Pakistan.
[6] Egyptian Atomic Nuclear Research Center, Inshas, Egypt.
[†] Correspondince should be addressed to J.L. (jie.luo@mail.Hzau.edu.cn).

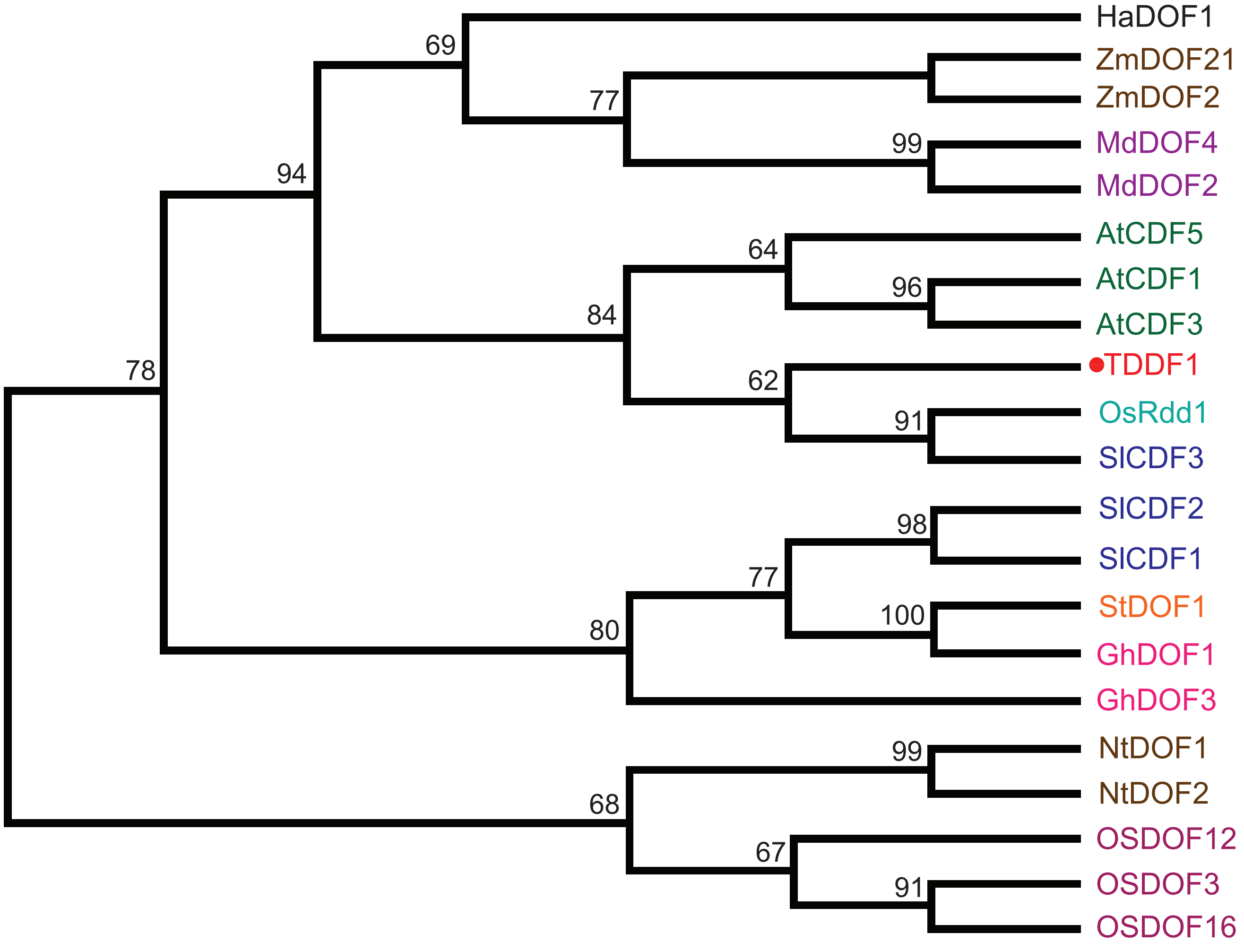

Fig. S1 phylogenetic analysis of *TDDF1*. The neighbor-joining tree was constructed using aligned amino acid sequences. Bootstrap values from 1,000 replicates are indicated at each node.

a

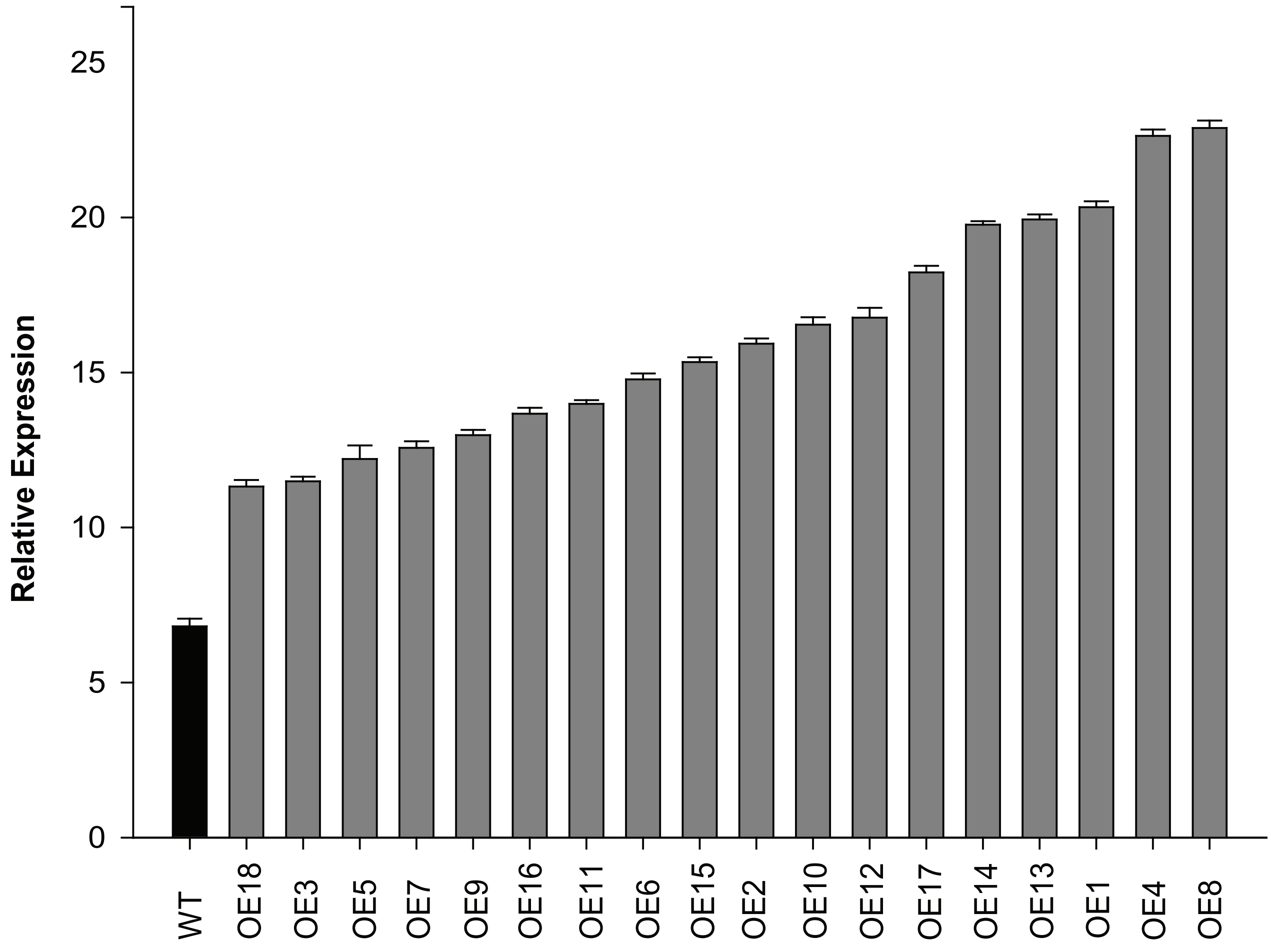

b

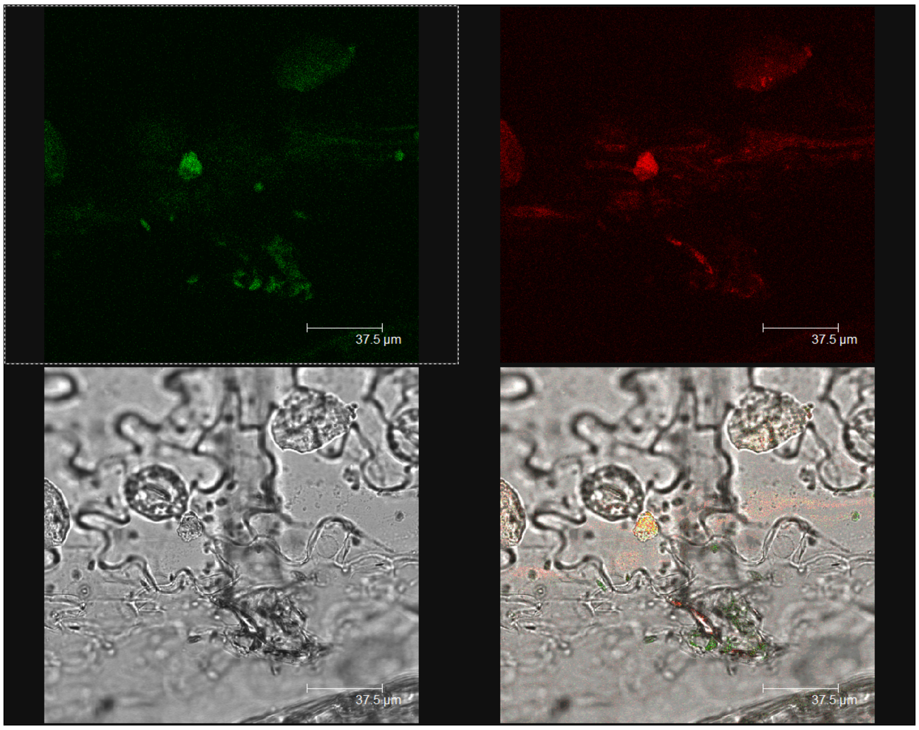

Fig. S2 (a) Transcript levels of *TDDF1*, in wild-type and 18 over-expression lines. Transcript level was determined by real-time quantification RT-PCR. (b) Subcellular localization of *TDDF1* in tobacco leaves. *TDDF1* signals were mostly overlapped with GFP (up-left), TDDF1-RFP (up-right), bright field (down-left), and merged image (down-right) were presented. Bar =25.85 μm.

```
+ GATCACTTTA GAAGGAGAAG TTACACAAAG CAAACAAGTT GATGAACTTG TTGATCCAAC TGCAGACTCG
- CTAGTGAAAT CTTCCTCTTC AATGTGTTTC GTTTGTTCAA CTACTTGAAC AACTAGGTTG ACGTCTGAGC

+ TCGATTGAAC CAGAAACATC ATCTGGTATA AGCGATGACA TCAAGATGCA GGACGCGGAT AAAGAGACGT
- AGCTAACTTG GTCTTTGTAG TAGACCATAT TCGCTACTGT AGTTCTACGT CCTGCGCCTA TTTCTCTGCA

+ TATCATCAAA ATCTATCGAG GAGGAGGATT CGAGTGAGGA GAAGGCACTC AAGAAGCCTG ATAAATTGAT
- ATAGTAGTTT TAGATAGCTC CTCCTCCTAA GCTCACTCCT CTTCCGTGAG TTCTTCGGAC TATTTAACTA

+ TCCATGTCCG CGATGTAATA GCATGGAAAC AAAGTTCTGT TATTACAATA ATTACAACGT CAATCAGCCT
- AGGTACAGGC GCTACATTAT CGTACCTTTG TTTCAAGACA ATAATGTTAT TAATGTTGCA GTTAGTCGGA

+ CGTTACTTCT GCAAGAACTG CCAGAGATAT TGGACTGCTG GAGGTACAAT GAGAAATGTG CCTGTGGGAT
- GCAATGAAGA CGTTCTTGAC GGTCTCTATA ACCTGACGAC CTCCATGTTA CTCTTTACAC GGACACCCTA

+ CTGGTCGTCG TAAGAACAAG AGTTCTTCCA TATCGAATTA TCCTCTTCAA GCAGGTCGAG TCGAAGCAGC
- GACCAGCAGC ATTCTTGTTC TCAAGAAGGT ATAGCTTAAT AGGAGAAGTT CGTCCAGCTC AGCTTCGTCG

+ AGCTCACGGA ATGCATCTTC CTGCTTCAAG GACAAATGGA ACTGTCCTTA CATTTGGATC AGATAAACCC
- TCGAGTGCCT TACGTAGAAG GACGAAGTTC CTGTTTACCT TGACAGGAAT GTAAACCTAG TCTATTTGGG

+ CTTTGTGACT CTATGGTTTC TGCATTGAAC TTAGCTGAGA ATTCACATAA TATGAATCGA AATGAATACC
- GAAACACTGA GATACCAAAG ACGTAACTTG AATCGACTCT TAAGTGTATT ATACTTAGCT TTACTTATGG

+ ATGGATCGGA ACGAAGAATG CCTACAATCG GGAATGATCA ATCAAATGGA AGTTGTAGTA CAGCTTCAAG
- TACCTAGCCT TGCTTCTTAC GGATGTTAGC CCTTACTAGT TAGTTTACCT TCAACATCAT GTCGAAGTTC

+ TGTAACTGAC AAAGAAAGCA GTGCTGGTAC TCATGATTTA GCTAATTGGA ATAATTTCCA GCCATTTCCT
- ACATTGACTG TTTCTTTCGT CACGACCATG AGTACTAAAT CGATTAACCT TATTAAAGGT CGGTAAAGGA

+ CCTCAAGTAC CCTACTTTCA GGGTGCTCCG TGGCCTTATT CTGGCTTTCC AGTATCATTC TATCCAGCAG
- GGAGTTCATG GGATGAAAGT CCCACGAGGC ACCGGAATAA GACCGAAAGG TCATAGTAAG ATAGGTCGTC

+ CACCGTACTG GGGATGCACC GTGCCAAACC CTTGGAACGT ACCTTGGCTT TCATCCGATC AATCAGTCCA
- GTGGCATGAC CCCTACGTGG CACGGTTTGG GAACCTTGCA TGGAACCGAA AGTAGGCTAG TTAGTCAGGT

+ TAACAACAGT CCTACCTCAC CGACATTAGG AAAACATTCT CGAGATGAAA GCAAGCTTGA TCCATCACAA
- ATTGTTGTCA GGATGGAGTG GCTGTAATCC TTTTGTAAGA GCTCTACTTT CGTTCGAACT AGGTAGTGTT

+ TCAAGGAGAA GAGATACTAC TTTGCAGGAT AGAGAAGGGG AGAGATGTGT ACTGATTCCG AAGACATTAA
- AGTTCCTCTT CTCTATGATG AAACGTCCTA TCTCTTCCCC TCTCTACACA TGACTAAGGC TTCTGTAATT

+ GGATTCATGA TCCAAATGAA GCGGCTAAAA GCTCTATATG GTCAACACTA GGTATCAGGA ATGAGAAGAT
- CCTAAGTACT AGGTTTACTT CGCCGATTTT CGAGATATAC CAGTTGTGAT CCATAGTCCT TACTCTTCTA

+ TGATTCGACT CGTGGTACAA TGCTCTTCAG TGCCTTTAAT CCAAAAGCTG ATCATAGAAA TCGCGAACTT
- ACTAAGCTGA GCACCATGTT ACGAGAAGTC ACGGAAATTA GGTTTTCGAC TAGTATCTTT AGCGCTTGAA

+ GACACTTCTT TTGCCTTGCA AGCTAATCCA GCAGCCTTGT CTAGATCACT TCATTTTCGT GAGAGTACAC
- CTGTGAAGAA AACGGAACGT TCGATTAGGT CGTCGGAACA GATCTAGTGA AGTAAAAGCA CTCTCATGTG

+ GATGATATAA GACTTACAAC ATGGTTCATG GTTGCCTAGT ACCAATCATT CATGTTAAGT CTCTTGCATA
- CTACTATATT CTGAATGTTG TACCAAGTAC CAACGGATCA TGGTTAGTAA GTACAATTCA GAGAACGTAT

+ AAATGCAGGA TAAAGCTGCG TACAGTTGAC CTTTGTGGAC TGGCCCTTCC TCGGACCCTG CGCATAGCGG
- TTTACGTCCT ATTTCGACGC ATGTCAACTG GAAACACCTG ACCGGGAAGG AGCCTGGGAC GCGTATCGCC

+ GTTCTTGTGT CTAGGAGGAG AAGGAGACAG ATGCATAGTG TCTACAAGTT TTATAGTTTT GCAGTACTAT
- CAAGAACACA GATCCTCCTC TTCCTCTGTC TACGTATCAC AGATGTTCAA AATATCAAAA CGTCATGATA

+ GTGTACCTTG TACATAGACC TGTAGAGTTA TATTTAATAG ATAGAGATAA ATAAGATGCA GCTGAAGTTT
- CACATGGAAC ATGTATCTGG ACATCTCAAT ATAAATTATC TATCTCTATT TATTCTACGT CGACTTCAAA

+ AATCTCTAGA GGATCCCCGG GTACCGAGC
- TTAGAGATCT CCTAGGGGCC CATGGCTCG
```

Fig. S3. *Cis*-acting element analysis of *TDDF1* promoter. AE-box (AGAAACAT), part of a module for light response. ARE-box (AGAAACAT), *cis*-acting regulatory element essential for the anaerobic induction. ATCT-motif (AATCTAATCC), part of a conserved DNA module involved in light responsiveness. BOX III (CATTTACACT), protein binding site. BOX-W1 (TTGACC), fungal elicitor responsive element. CAAT-box (CAAT), common *cis*-acting element in promoter and enhancer regions. CGTCA-motif (CGTCA), *cis*-acting regulatory element involved in the MeJA responsiveness. G-box (GCCTTGTGTAG), *cis*-acting regulatory element involved in light responsiveness. GAG-motif (AGAGATG), part of a light responsive element. Ibox (CCATATCCAAT), part of a light responsive element. MBS (CAACTG), MYB binding site involved in drought-inducibility. O2-site (GATGACATGA), cis-acting regulatory element involved in zein metabolism regulation. Skn-1_motif (GTCAT), cis-acting regulatory element required for endosperm expression. TATA-box (TATA), core promoter element around -30 of transcription start. TCT-motif (TCTTAC),part of a light responsive element. TGACG-motif (TGACG), cis-acting regulatory element involved in the MeJA-responsiveness. Circadian (CAANNNNATC), *cis*-acting regulatory element involved in circadian control.

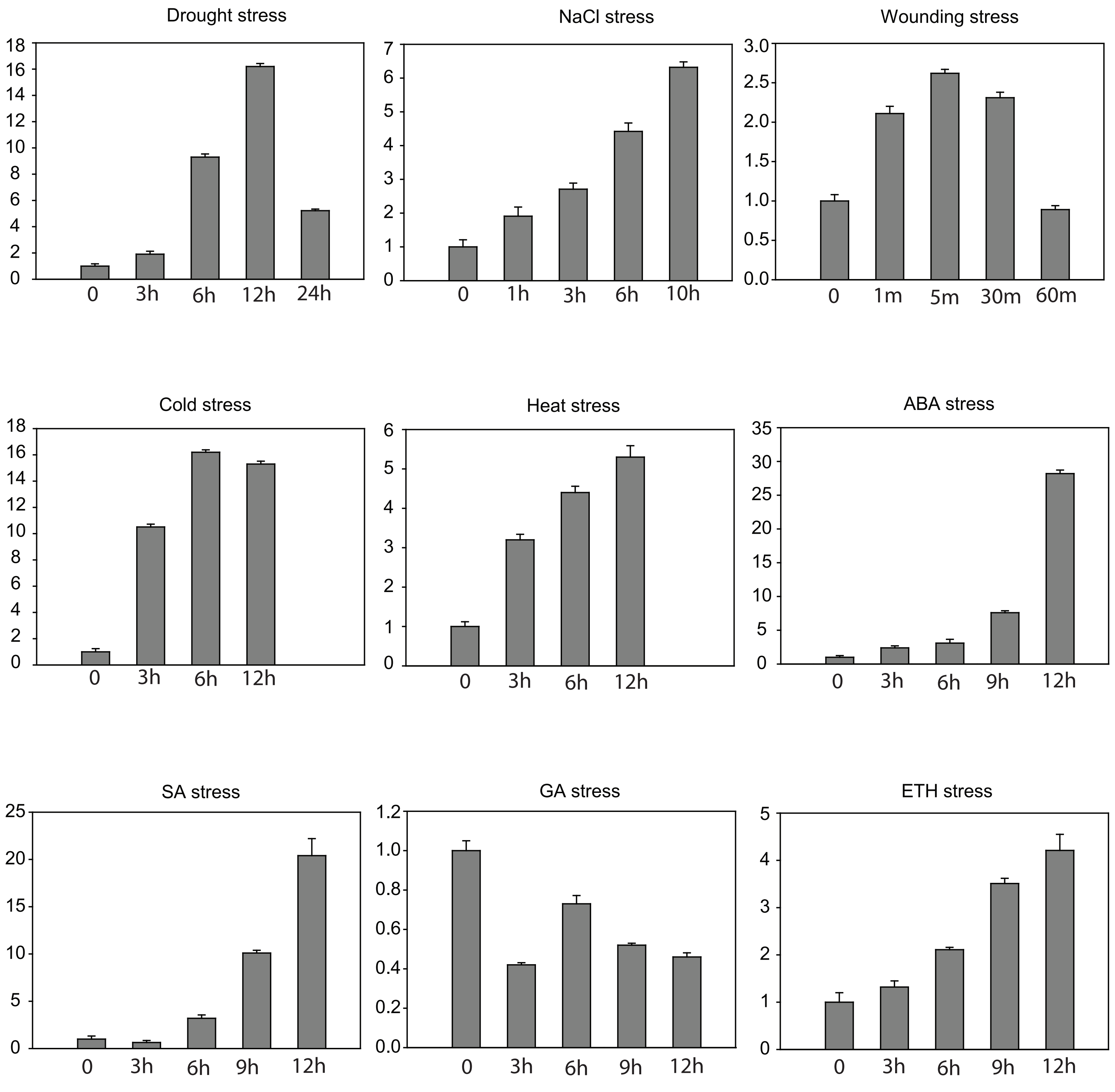

Fig. S4 Expression levels of *TDDF1* in tomato leaves under phytohormones and different stress conditions. The leaves of 2 -month old plants were used for RNA extraction in wild-type plants after treatment with drought, 200mM NaCl, wounding, °C cold, 40 °C heat, 100 μM ABA, 100 μM SA, 100 μM GA, and 100 μM ETH, respectively. All samples were collected at the indicated time points ('h' and 'm' refer to hours and minutes after treatment, respectively) from three biological replicates. Single (*P <0.05) and double (**P <0.01) asterisks denote statistically signifcant differences between the stress treatment and the 0h control. Actin gene was used as an internal control in the qRT-PCR.

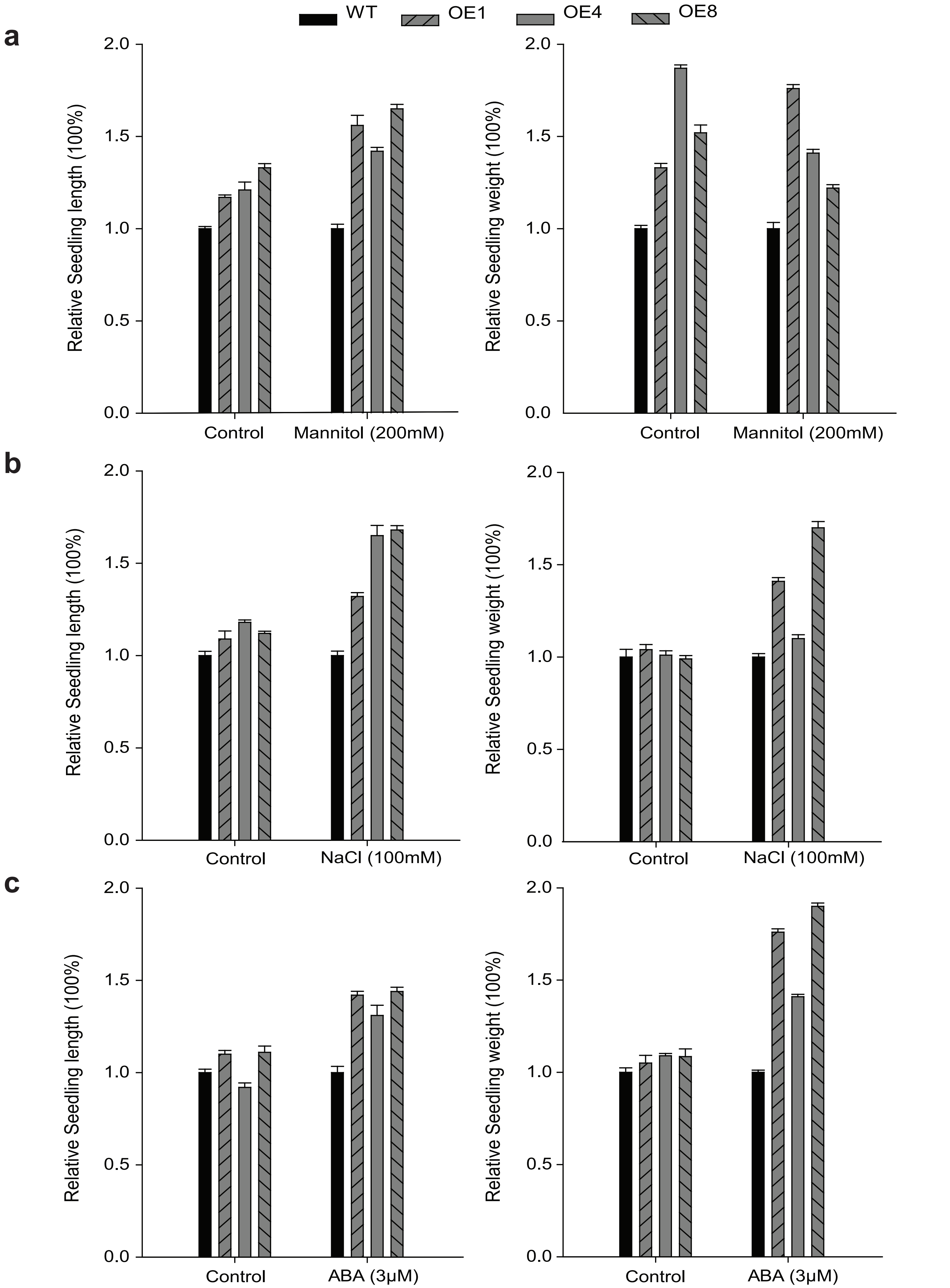

Fig. S5 Growth performance of *TDDF1* OE and wild-type seedling treated with mannitol, salt, and ABA stresses. Seedling length and weight of transgenic and wild-type lines after treatment with 200mM mannitol (a), 100mM NaCl (b), 3 μM ABA (c), and without stress as control. The seedling were grown in half-strength MS medium. the data shown are the mean±SE (n=6). Single (**P <0.05*) and double (***P <0.01*) asterisks denote statistically significant differences between transgenic and wild-type lines.

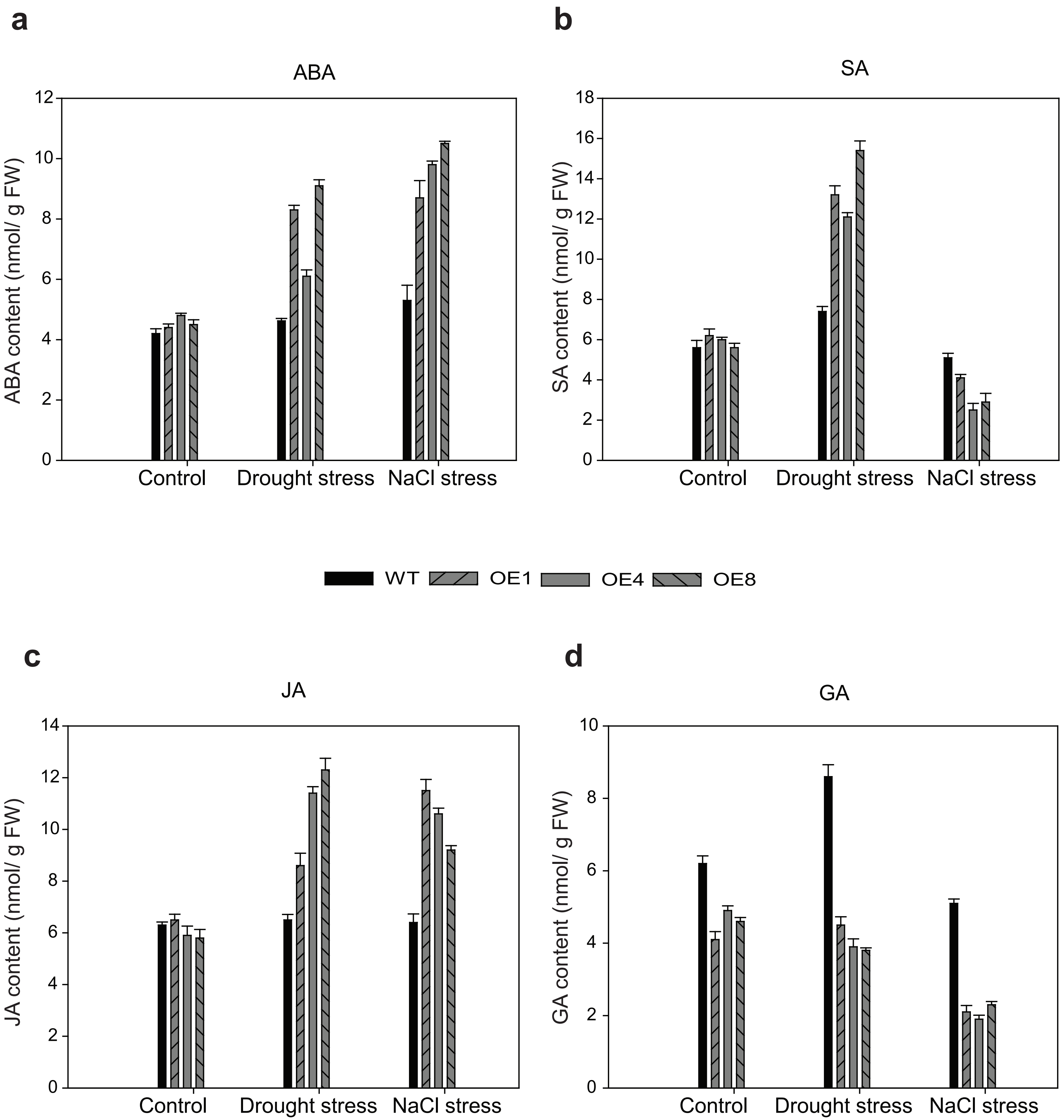

Fig. S6 Endogenous ABA, SA, JA, and GA contents in *TDDF1* OE and wild-type 2 month-old plants measured by HPLC. ABA content (a), SA content (b), JA content (c), and GA content (d), after withholding water for 7 days as drought stress, subjected to 100mM NaCl as salt stress, and without stress as control. Variance analysis was performed to determine significant differences (*$P < 0.05$ and **$P < 0.01$) between the WT and TDDF1 OE lines.

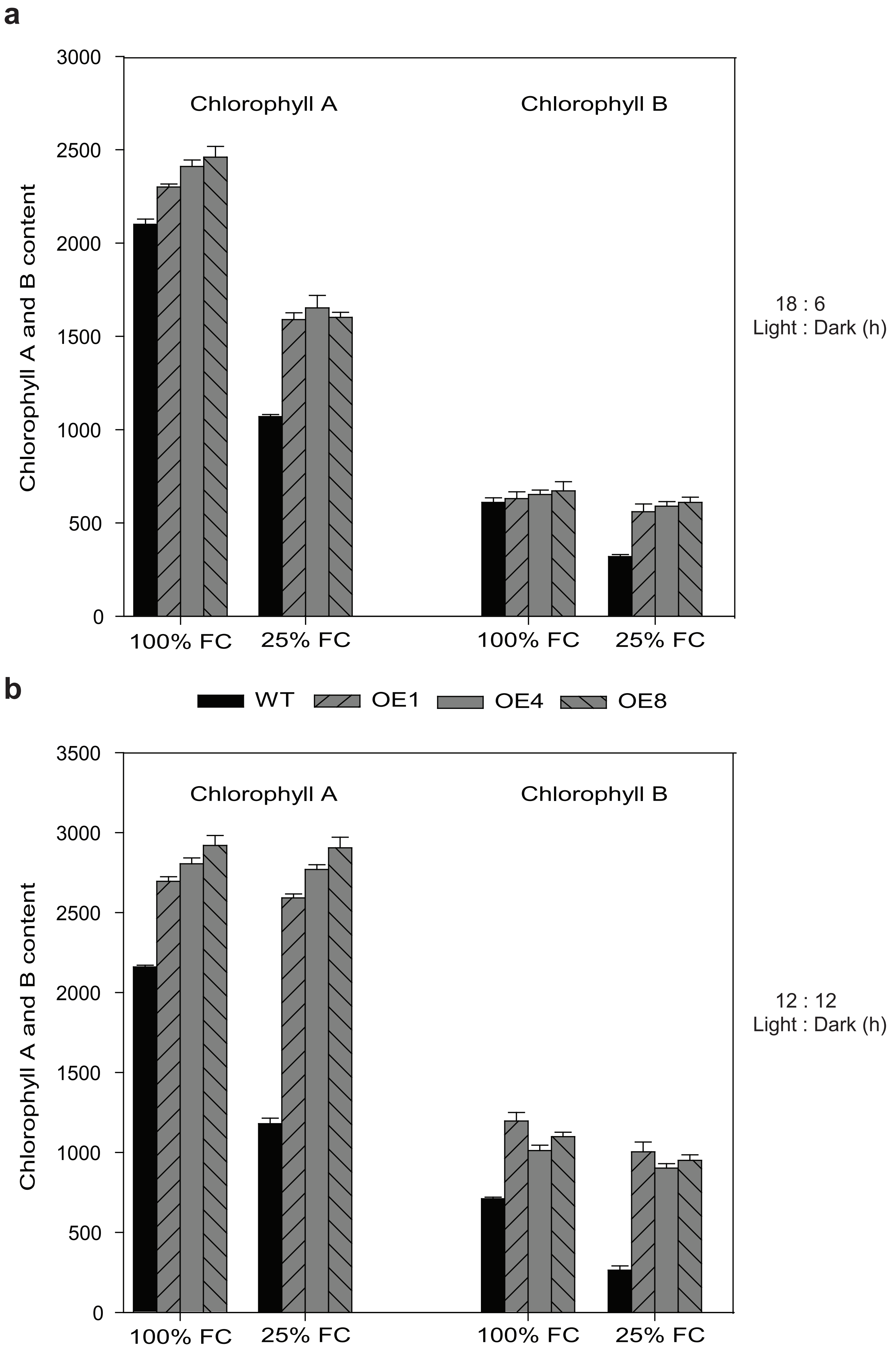

Fig. S7 *TDDF1* overexpression enhances Chlorophyll A and B synthesis in tomato under 25% FC as drought stress or 100% FC as control. The Chlorophyll A and B content were measured in wild type and transgenic plants grown under (a) (16:6 Light: Dark); (b) or (12:12 Light: Dark) coditions. Means values (± SD) are shown (*n*=3).

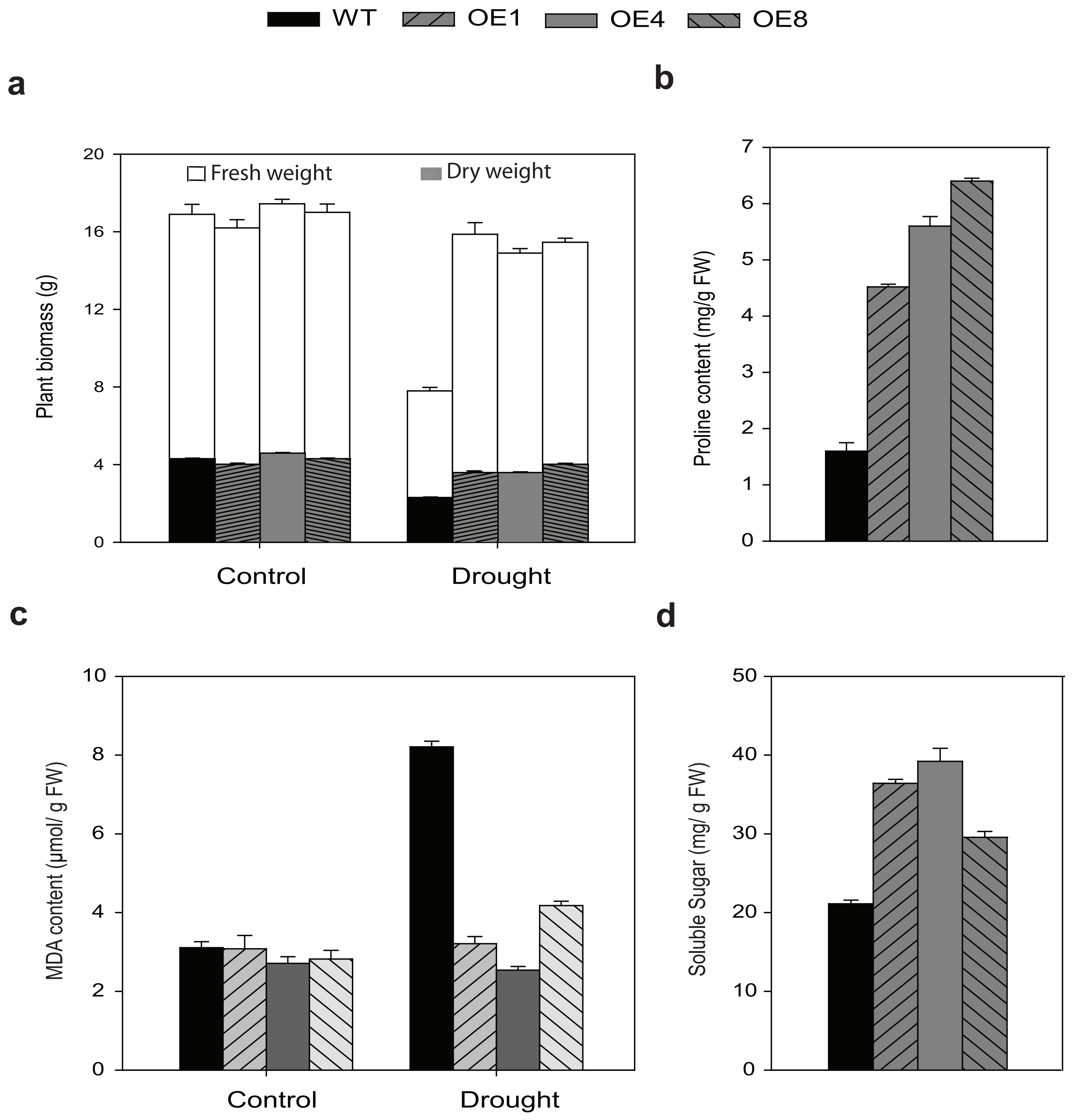

Fig. S8 *TDDF1* overexpression enhances drought tolerance in tomato. Effect of drought on fresh and dry weights of *TDDF1*- OE and wild-type lines (a); Proline accumulation in plant leaves under drought stress or normal condition (b); MDA content in plant leaves under drought stress or normal condition as control (c); Soluble sugar content in plant leaves under drought stress (d). Means values (± SD) are shown (*n*=3).

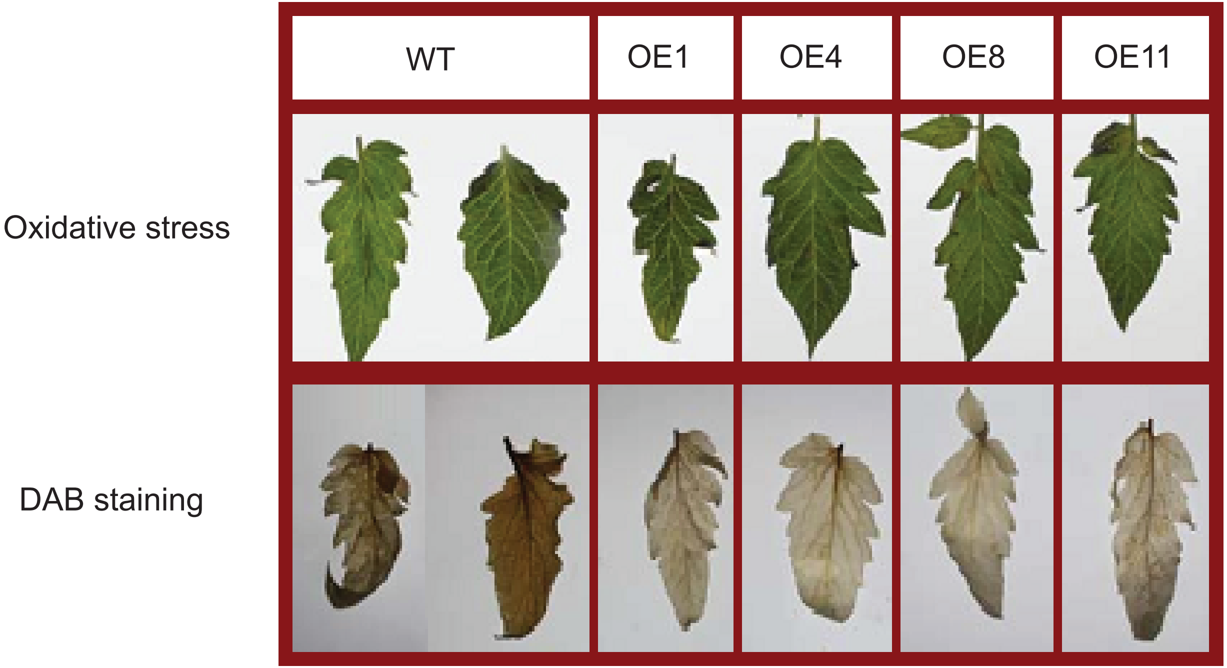

Fig. S9 Oxidative stress assay on the leaves of transgenic and wild-type plants. The leaves of *TDDF*1-OE and wild type plants were detached and sprayed with 100 μM paraquat for 24h (up), and the phenotype of DAB staining (down).

Table S1. Primers sequences used for functional and expression analysis in this study.

primer name	forward primer sequence (5'-3')	reverse primer sequence (5'-3')
Full-length *TDDF1* cDNA	CCACTTGTGGGATTACACACC	AAACTTCAGCTGCATCTTATTTATCTC
TDDF1 for real time PCR	ACGAAGAATGCCTACAATCGGGAAT	ACGGTGCTGCTGGATAGAATGATACTG
DREB2 for real time PCR	AAACGGGCTCGCCCCCAAAACCCC	AATTGGGAGTGAATTCGGTGAGTA
MAPK2 for real time PCR	ATTCCACCAACCTCAACGA	TGCTAGGCTTCAAGTCCC
PR1 for real time PCR	AGGTGACACTATAGAATAAACAAT	GTACGACTCACTATAGGGATAGCAAC
SFT for real time PCR	AACTCGAGGTCATGCCTAGAGAACGTG	AAGGATCCTCAATCAGCAGATCTTCTACG
SlCDF1 for real time PCR	GTAATTATTCAAGTGGTGGGC	TTACCAAGATCCTCCAGTACC
SlCDF2 for real time PCR	GTAGCCGCAAAAATGCAAAAC	CAGGCCCATGAGAACTCAACA
SlCDF3 for real time PCR	TGATGATCCAGGAGAGGCTGC	TTATAAGCTCTCATTGCAAAGGC
NCED3 for real time PCR	TGGTTTTCATGGGACATTCATTAG	ATCTCCCTTCTCAACTCAACTCCCT
PAL1 for real time PCR	CGAGCAGAGAGCGACGGCCG	GGCTTCTCAGTGGATGATATTGG
OPR3 for real time PCR	CGAGCAGAGAGCGACGGCCG	GGCTTCTCAGTGGATGATATTGG
HEML for real time PCR	AAGAAAACCCATTGCAGCAC	AGACATGGAGCACCGAAACT
HEMB1 for real time PCR	CAGTTGTGCAAACAGGCAGT	GCTGAAATCCTTCAGCATCA
HEMC for real time PCR	CAGGTTCCCTGGCTGAACT	CAACACATTGAGTGAGGGATAG
HEME1 for real time PCR	GTGGACAACTCCCACCTCAT	CCGTTCAAGAAGTCCACCAT
HEMG1 for real time PCR	CATACTTTTCCACCTCCAAGCG	GTCAACTTCTGAGGGAGGAAC
CHLD for real time PCR	GGAAGCGTGGAACAGCTAAA	AGCTAGCCCATCTTCCCACT
CHLM for real time PCR	GACCGGTTGTTTGTCGATTC	ACTCCTCTTGCGCCTGTTT
CRD for real time PCR	TCTCGCTTCTTCTGCCTTTC	AGTTGTGCGGTTTGTCTCAA
CAO for real time PCR	CCTTTCTCTTTGTGCCGCTCT	CCTCCTCCCCATACACAGC
Actin for RT-PCR	ATGGCAGACGGAGAGGATATTCA	GCCTTTGCAATCCACATCTGCTG
Actin for real time PCR	GTCCTCTTCCAGCCATCCAT	ACCACTGAGCACAATGTTACCG
